# Water Networks in Photosystem II Using Crystalline Molecular Dynamics Simulations and Room-Temperature XFEL Serial Crystallography

**DOI:** 10.1021/jacs.3c01412

**Published:** 2023-06-27

**Authors:** Margaret D. Doyle, Asmit Bhowmick, David C. Wych, Louise Lassalle, Philipp S. Simon, James Holton, Nicholas K. Sauter, Vittal K. Yachandra, Jan F. Kern, Junko Yano, Michael E. Wall

**Affiliations:** † Molecular Biophysics and Integrated Bioimaging Division, Lawrence Berkeley National Laboratory, Berkeley, California 94720, United States; ‡ Computer, Computational and Statistical Sciences Division, Los Alamos National Laboratory, Los Alamos, New Mexico 87545, United States; § Center for Non-linear Studies, Los Alamos National Laboratory, Los Alamos, New Mexico 87545, United States; ∥ Department of Biochemistry and Biophysics, University of California, San Francisco, San Francisco, California 94158, United States; ⊥ SSRL, SLAC National Accelerator Laboratory, Menlo Park, California 94025, United States

## Abstract

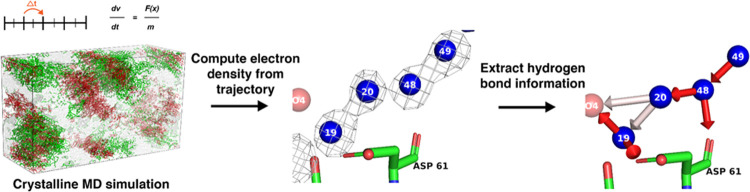

Structural dynamics of water and its hydrogen-bonding networks play an important role in enzyme function via the transport of protons, ions, and substrates. To gain insights into these mechanisms in the water oxidation reaction in Photosystem II (PS II), we have performed crystalline molecular dynamics (MD) simulations of the dark-stable S_1_ state. Our MD model consists of a full unit cell with 8 PS II monomers in explicit solvent (861 894 atoms), enabling us to compute the simulated crystalline electron density and to compare it directly with the experimental density from serial femtosecond X-ray crystallography under physiological temperature collected at X-ray free electron lasers (XFELs). The MD density reproduced the experimental density and water positions with high fidelity. The detailed dynamics in the simulations provided insights into the mobility of water molecules in the channels beyond what can be interpreted from experimental *B*-factors and electron densities alone. In particular, the simulations revealed fast, coordinated exchange of waters at sites where the density is strong, and water transport across the bottleneck region of the channels where the density is weak. By computing MD hydrogen and oxygen maps separately, we developed a novel Map-based Acceptor–Donor Identification (MADI) technique that yields information which helps to infer hydrogen-bond directionality and strength. The MADI analysis revealed a series of hydrogen-bond wires emanating from the Mn cluster through the Cl1 and O4 channels; such wires might provide pathways for proton transfer during the reaction cycle of PS II. Our simulations provide an atomistic picture of the dynamics of water and hydrogen-bonding networks in PS II, with implications for the specific role of each channel in the water oxidation reaction.

## 1 Introduction

Water enables the chemistry of life. It is the medium in which all biological reactions take place, it controls the kinetics and energetics of molecular encounters, and it also serves as a substrate for proton transport. The mobility and flexibility of water in forming hydrogen-bond networks are central to these roles; however, our understanding of how water dynamics enable and control biological function is far from complete. Attempts to visualize the chemical and structural changes of water molecules in real time have yielded key insights, while also revealing technical and methodological challenges that must be overcome to increase our understanding.^1,2^

X-ray crystallography has been the main experimental tool for understanding protein structure along with the location of waters and the networks they form. Neutron crystallography also has been used, in particular to identify the location of hydrogen nuclei. Recently, cryo-electron microscopy has become a mainstream approach for visualizing single molecules. After deriving a starting model from the structures collected through these techniques, computational methods like molecular dynamics (MD) simulations have provided insight into the atomic-scale interactions in proteins, which has led to better modeling of water in biomolecules.

For macromolecular crystallography, the introduction of X-ray free electron lasers (XFELs), which generate ultrabright X-ray pulses by accelerating relativistic electrons through a periodic arrangement of undulator magnets, has enabled us to look at time-resolved structural changes of enzymes. The data can be collected at physiological temperature prior to the onset of X-ray-induced changes, by taking snapshots after triggering reactions with a wide array of methods.^3−9^ The effects of temperature on protein structure and dynamics have been investigated extensively, with the implication that cryocooling could modify the conformational state of enzymes and alter the water network.^10−18^ Thus, by determining the 3D structure of a protein under physiological temperatures and at timepoints along the reaction pathway, XFEL crystallography allows us to capture the most biologically and chemically relevant conformational states of a protein.

Concurrently, advances in MD methods and high-performance computing have enabled >100 ns MD simulations (such as the one discussed in this paper) for protein systems with 10^5^–10^6^ atoms to be performed routinely. Recently, crystalline MD simulations have been utilized in X-ray diffuse scattering studies as well as for closing the “*R*-factor gap” in proteins (the gap in agreement between observed and predicted structure factors, i.e., *R*_work_ and the experimental errors i.e., *R*_merge_) by modeling the protein–solvent interaction.^19−23^ In these studies, the crystalline conditions were simulated by using the known crystal packing and experimental sample conditions to generate a periodic simulation box that is consistent with the unit cell dimensions. Using this scheme enables the simulations to be compared directly to crystallographic data using standard crystallography methods available, e.g., in Phenix^24^ and CCP4.^25^ In addition to using standard methods, it is also possible to use simulated diffraction images, produced from crystalline MD trajectories, to make comparisons.^26^

**Figure 1:**
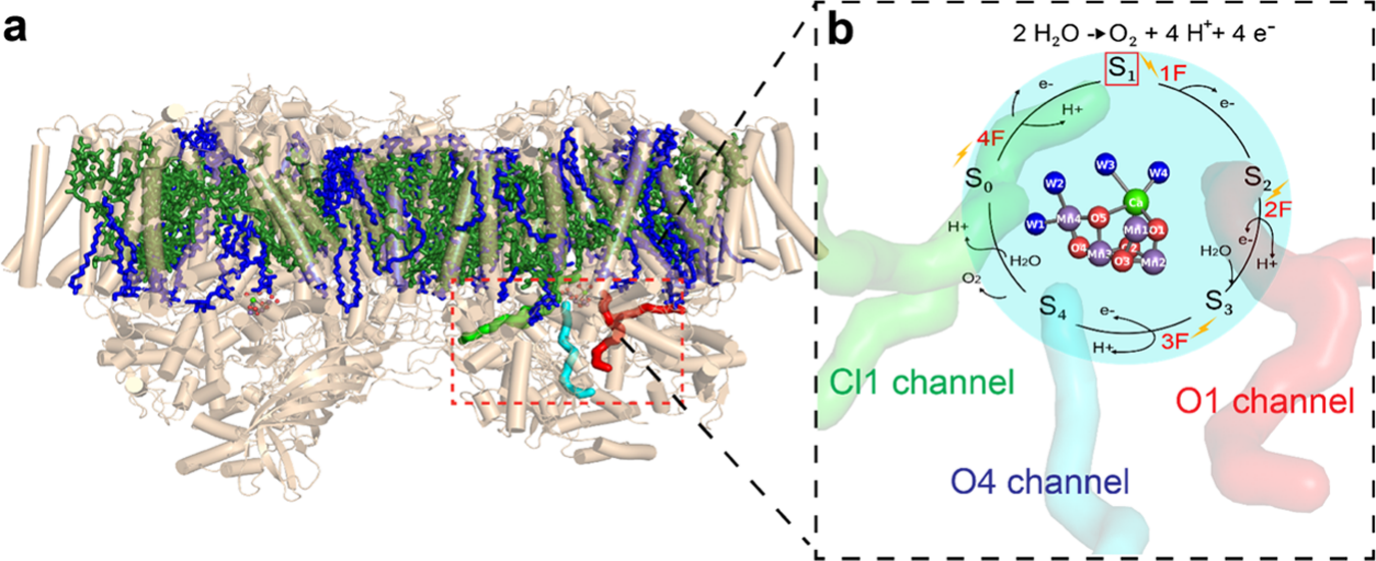
Overview of PS II and its electron donor site where water is oxidized to molecular oxygen. (a) The protein structure of PS II from *Thermosynechococcus vestitus* serves as the starting point for our MD model. The lipids (blue) are highlighted along with cofactors (green) and the three water channels (O1, pink; O4, cyan; Cl1, green). (b) OEC (only the Mn_4_CaO_5_(H_2_O)_4_ cluster) is shown in the context of the water channels, along with the Kok S-state cycle, advancing through the S-states (S*_i_*, *i* = 1–4) by illumination with 1 through 4 flashes (1–4F). The S_1_ state (solid red box) is the focus of the current work.

Here, we combine XFEL crystallography data with crystalline MD simulations to investigate water structure, dynamics, and hydrogen-bonding networks in Photosystem II (PS II), the enzyme responsible for catalyzing the light-induced oxidation of water to molecular oxygen.^27,28^ Water serves a dual role as both substrate and solvent in PS II. The active site for PS II is a metal cluster called the oxygen-evolving complex (OEC; Mn_4_CaO_5_), which is located on the lumenal side of the protein complex. The OEC is shielded from the direct exposure to bulk solvent by large extrinsic loops of the D1, D2, CP43, and CP47 subunits as well as by several membrane extrinsic subunits—PsbO, PsbU, and PsbV in the case of the thermophilic cyanobacterial complex. However, the cluster is connected to the bulk solvent via hydrophilic channels (Figure 1a).^29−32^ The formation of molecular oxygen from two water molecules at the cluster takes place in a sequence of four intermediate steps called the Kok cycle (Figure 1b), starting from the dark resting S_1_ state.^33,34^ Each photon absorption by the chlorophylls in the PS II reaction center (P680; not shown in the figure) leads to oxidation of the cluster and further advancement along the cycle (S_*i* ∈ {0,1,2,3,4}_) until the transient S_4_ state is reached at which point molecular oxygen is formed and the cluster is reset to the S_0_ state for the process to begin again.

Both water transport and the proton release, in a well-controlled stepwise manner, are critical for the water oxidation reaction, as it advances through the S-state intermediates in the Kok cycle. Bulk water proximal to the catalytic center is detrimental as the intermediates that are generated with the high oxidative potentials present at the catalytic center can lead to very reactive species that can damage the protein. Thus, it is important that the water is delivered to the catalytic center in a stepwise and controlled manner, and following this process is critical for understanding the mechanism of water oxidation by PS II. Several channels in PS II have been identified using many experimental studies and simulations.^29−32,35−40^ The three which are thought to be important for water and proton transport are the O1 channel (“large channel”), the O4 channel (“narrow channel”), and the Cl1 channel (Figure 1). All three channels start near the OEC and extend outward toward the lumenal side of the complex to the bulk water. The O1 channel (proposed water channel, near the O1 bridging ligand of the Mn_4_CaO_5_ complex)^8,32^ originates near Ca in the OEC before splitting into two branches, A and B. The O4 channel (proposed proton channel in the S_0_ to S_1_ transition, near the O4 bridging ligand of the Mn_4_CaO_5_ complex)^40^ starts by the O4 side of the OEC and extends toward the subunits PsbO and PsbU without branching. Finally, the Cl1 channel (proposed proton channel at least in the S_2_ to S_3_ transition, Cl^–^ binding site is located near this)^8,41−43^ originates near Mn4 and contains both a short branch (A, sometimes known as the broad channel)^29^ and a long branch (B, extends toward PsbO). Further details on these channels have been discussed in Hussein et al.^8^

To better understand the involvement of the water networks of PS II in the transport of water and the transfer of protons through the channels, we prepared a crystalline MD model of the PS II unit cell in explicit water, using a physiologically relevant room-temperature XFEL crystal structure in the dark-stable S_1_ state. While simulating proton transfer itself requires a quantum mechanical simulation, here we only look to infer the likely pathways of proton transfer from the hydrogen bond networks derived from classical MD simulation. Doing a crystalline MD simulation with our protocol also allows us to reproduce the experimental water structure and cover a much longer timescale and larger system size than would be possible using a quantum simulation.

We carried out the simulation using two different ionic solvent conditions. In the first case, we added 479 Na^+^ ions to neutralize the negative charge of the protein. In the second case, we added Na^+^ and Cl^–^ ions in equal proportion (489 ions each) to mimic experimental buffer conditions of the XFEL crystallography, and then an additional 479 Na^+^ ions to neutralize the negative charge.^8^ We refer to the former in the text as the “simulation” and the latter as the “Cl^–^ simulation”. The comparison of these conditions enabled us to investigate the transport of ions—which cannot be resolved experimentally—as well as electrostatic effects of elevated salt levels.

In contrast to previous solution simulations of PS II,^36−38,40,44^ performing MD simulations in the crystalline state enables the construction of MD electron density maps that can be directly compared to the room-temperature XFEL diffraction data.^7,8^ Such a comparison has recently been carried out by Wych et al. on the catalytic subunit of mouse protein kinase A (PKA-C) leading to a new solvent model and an improved protein structure including alternative conformations.^45^ The construction of electron density maps follows the procedure outlined in Wall et al.^46^ In addition to calculating total density maps for the waters in the system, we decomposed the water density into hydrogen and oxygen components. This helps us infer information about hydrogen bonding and water dynamics in the channels that have not been accessible previously. We developed a new technique called MADI (Map-based Acceptor–Donor Identification) for this purpose. The MADI analysis yields information about both the strength and directionality of the inferred hydrogen bonds leading to a clear picture of the network connecting the Mn cluster to the bulk. Using our methods, we are able to make several key observations pertaining to the water transport and hydrogen bond network and to connect them to the functioning of the PS II enzyme.

## 2 Methods

### 2.1 Molecular Model and Force Field Parameters

The protein crystal MD model was prepared from the X-ray diffraction structure of PS II from *Thermosynechococcus vestitus* (PDB ID Code 7RF2), collected at the Linac Coherent Light Source (LCLS) at SLAC National Accelerator Laboratory and SACLA in Japan. Since our S_1_ state data has a resolution of 2.08 Å, water positions could be modeled in the electron density with high accuracy. Details of the refinement have been described elsewhere.^7^ Starting from the crystal structure, we used the P2_1_2_1_2_1_ space group to expand the asymmetric unit to the P1 unit cell with UCSF Chimera, resulting in four copies of the PS II dimer in the unit cell (with unit cell dimensions *a* = 116.92, *b* = 221.63, *c* = 307.83 Å, α = β = γ = 90°). The coordinates for 13 types of nonstandard lipids/cofactors/ions were extracted from the expanded PDB file, and positions for their hydrogen atoms were generated using the LEaP program.^47^ Molecular model information (.*mol*2) and force field modification files (.*frcmod*) from Sakashita et al.^44^ were used for a majority of the lipids/cofactors/ions (**OEX, PHO, HEM, LHG, LMG, PL9, BCR, CLA, DGD, W1–W4**) to generate the topology (.*itp*) and structure (.*gro*) files. For the remaining lipids/cofactors/ions (**SQD, STE, FE, CL**), .*mol*2 files were generated using UCSF Chimera and parameterized with parmchk tools^47,48^ (see Tables S1–S3). The topology file for the OEC was based on the one used by Sakashita,^44^ however, a few modifications were made in order to preserve the geometry of the Mn cluster. Specifically, the bond lengths were adjusted to match those in our S_1_ state structure^4^ and the angle force constants were increased by a factor of 4.17 to prevent the Mn4 atom from drifting.

No nonstandard protonation states were used in this work. For a table specifying net charges on all lipids/cofactors/ions, see Table S4. Additionally, relevant topology files can be found at https://github.com/mdoyle17/Molecular-Dynamics-Analysis-in-Python. Protein residues were parameterized with the AMBER14SB force field. Hydrogens for the protein atoms were added through GROMACS by pdb2gmx. The parameterized system was then solvated with TIP3P waters using gmx solvate in GROMACS. Note that this solvent is in addition to the crystallographic waters which were already present in the model. The four waters ligated to the Mn_4_CaO_5_ cluster (W1, W2, W3, W4) were also modeled as H_2_O; however, they were treated as distinct ligands, and their residues were renamed to WON, WTW, WTH, WFO. They were not grouped in with the rest of the crystal waters so that the option of varying their protonation states remained open in the future.

It has been reported previously that crystalline simulations with protein and ligand restraints to the crystal structure allow for a good recall of crystallographic waters.^46^ With this information in mind, our protocol ensures that all heavy protein and ligand atoms are biased toward the crystal structure using harmonic restraints. The restraints are applied using springs that tether each atom to its position in the crystal structure. While the atoms are biased toward the crystal structure, they are still allowed to move—including adopting alternative conformations of side chains—if sufficiently favored by the MD force field. All ligand atoms have springs with a strength of 209.2 kJ/mol nm^2^ associated with them which is relatively moderate, and all protein heavy atoms (anything but hydrogen) have springs with a strength of 1000 kJ/mol nm^2^ applied to all heavy atoms to bias them toward the crystal structure. With the exception of the four waters ligated to the OEC (W1, W2, W3, W4) which experienced the same moderate restraint strength as other ligand atoms, all water atoms were unrestrained, enabling insights into water exchange dynamics from the results, as discussed in detail in the later sections.

### 2.2 MD Simulations

*GROMACS* version 2018 was used for the MD simulations.^49^ To avoid errors in computing mean structure factors due to changes in the unit cell, the simulations were performed using a constant volume (NVT ensemble), with fixed periodic box size. NVT simulation was performed using the Cori Supercomputer at NERSC using 20 nodes, 32 tasks per node at a rate of 11.668 ns simulation time per day of wall clock time. Our simulation was confined to a box with dimensions *a* = 117.56, *b* = 222.88, *c* = 309.90 Å, and α = β = γ = 90°, with the purpose of obtaining close packing conditions like those observed in crystals, resulting in a total volume of 8120 nm^3^. The simulation box was chosen to be slightly larger than the unit cell of the crystal to provide a small padding at the boundaries. Our buffer for this simulation consisted of just Na^+^ ions. We began by solvating the void volume of the crystal with 135 577 solvent molecules followed by 479 Na^+^ ions with the purpose of neutralizing the net charge on the protein. This was necessary as the particle-mesh-Ewald (PME) method, which was employed for computing long-range electrostatic interactions, requires charge neutrality for an accurate calculation.^50,51^ The water and ions were grouped separately from the protein and ligands for temperature coupling, as the former tends to generate more heating by force and integration errors.^49^

Next, an iterative solvent addition procedure for achieving near-atmospheric pressure in crystalline NVT simulations was applied. This involves rounds of energy minimization via the steepest descent algorithm followed by a 250 ps NVT simulation with simulated annealing, and then more solvation, until a mean pressure between −100 and 100 bar was achieved.^46^ The final model contained 861 894 atoms (81 471 of which belonged to lipids/ions/cofactors). A detailed breakdown of these atoms and their preparation may be found in Tables S1–S3, along with the copies per lipid/cofactor/ion.

To carry out the simulation, leap-frog integration with a time step of 1 fs was implemented. The output frequency for coordinates, velocities, and forces was set to 2 ps. Velocities were assigned from a Maxwell Boltzmann distribution at 300 K. The Lincs constraint algorithm, which resets bonds to their correct lengths after an unconstrained update, was used along with a Verlet cutoff scheme.^49^ Van der Waals and electrostatic cutoff distances were set to 1.4 nm. Periodic boundary conditions (PBC) were also employed to simulate an infinite crystalline system. Artificial correlations across the unit-cell boundary exist in such a simulation, but here we are mainly interested in using the simulations to compute the mean electron density, which we do not expect to be very sensitive to these correlations. Accurate studies of the large-scale dynamics would need to involve simulations of a 2 × 2 × 2 supercell or larger system,^23^ which is beyond the scope of the present study. In total, the simulation was run for 100 ns: The first 45 ns of which were used to ensure proper equilibration of the protein/lipids/cofactors with the solvent and thus were not used for further downstream processing. The trajectory from the final 55 ns of the simulation was extracted for statistical analysis.

A second simulation was carried out using a NaCl buffer instead of simply neutralizing the simulation volume using Na^+^ ions. For this, 489 waters were first replaced by Cl^–^ ions in order to achieve a 0.1M Cl concentration, just like in the experimental buffer. Next, 968 additional waters were replaced by Na^+^ ions in order to balance the charge of the system (note that this now includes balancing the original net negative charge on the protein as well as the negative charge introduced through the additional Cl^–^ ions). Everything else remained the same—and the new simulation was carried out for 100 ns. For identification purposes, this is referred to as “the Cl^–^ simulation.”

### 2.3 Mean Structure Factors

Mean structure factors *F*_MD_ were computed to 2.0 Å resolution for the last 55 ns of the MD trajectory and output as .mtz files using methods described previously,^23^ using xtraj.py in the *Lunus* software suite available at http://github.com/lanl/lunus. To summarize, *cctbx* methods are employed^52^ to enumerate over *N* snapshots. At each snapshot *i*, the *xray.structure.select()* method generates a new structure from only the selected atoms and then the *xray.structure.structure_factor()* method is used to calculate its structure factor *F*. Electron density maps were then calculated by averaging these structure factors and computing the fast Fourier transform (FFT). This process was done individually for all waters, water-oxygens, and water-hydrogens. Absolute scale maps were generated from each MD.mtz file by scaling the maps using cell volume and F000 of the selection when using the *fft* function from CCP4’s program suite.^25^ The result is an average electron density map for specific atom or molecule types.

### 2.4 Experimental XFEL Crystallography Data

The crystallography data for the dark-stable S_1_ state (PDB ID 7RF2) was collected at the MFX instrument of LCLS at the SLAC National Laboratory, Stanford, CA. The data was collected using a 40 fs X-ray pulse length at 9.5 keV with a pulse energy ranging between 2 and 4 mJ. The data was collected on the Rayonix-340HS detector with a 3 × 3 binning mode and with a 3 μm X-ray spot size. The Drop-on-Tape method was used for sample delivery.^53^

The XFEL data was processed with the DIALS software using the programs *dials.stills_process* for initial indexing/integration (using a target unit cell of *a* = 117.0 Å, *b* = 221.0 Å, *c* = 309.0 Å, α = β = γ = 90°, space group P2_1_2_1_2_1_), *cctbx.xfel.stripe_experiment* for ensemble refinement of the indexed lattices including unit cell and orientation refinement and finally *cxi.merge* for merging of the integrated reflections. Lattices that did not reflect beyond 3 Å were rejected. Images were integrated to the edge of the detector and a per-image resolution cutoff was used in the merging step. In total, 11734 lattices were merged to 2.08 Å (Final unit cell of *a* = 116.9 Å, *b* = 221.6 Å, *c* = 307.8 Å, α = β = γ = 90°, space group P2_1_2_1_2_1_).

Using the obtained merged intensities, initial model building was done using the high-resolution isomorphous 7RF1 structure as a template (1.89 Å) with rigid body refinement using *phenix.refine*. Subsequently, several alternate rounds of coordinate and *B*-factor refinement were carried out. Certain side-chain/backbone regions were also fixed in *Coot* using real space refinement. Custom restraints for the OEC were used that were based on available spectroscopic and chemical data. Custom restraints were also used for the Chlorophyll-a (CLA) and lipid molecules (STE). Waters were added in the later stages of refinement using the default criterion in *phenix.refine* as well as manually adding waters in certain regions that were identified using the 7RF1 structure. The reliability of water positions was checked by calculating polder omit maps (*phenix.polder*) and evaluating the peak height positions in those maps. Waters with peak height below 3σ were removed from the model. Final *R*-work/*R*-free values were 18.52/23.85%. Full details of the data collection and refinement for PDB id 7RF2 are available in Table S2 of Hussein et al.^8^ We also refer the reader to Table S3 of the same paper in order to match the water numbering used here with that in the deposited structure.

## 3 Results

**Figure 2:**
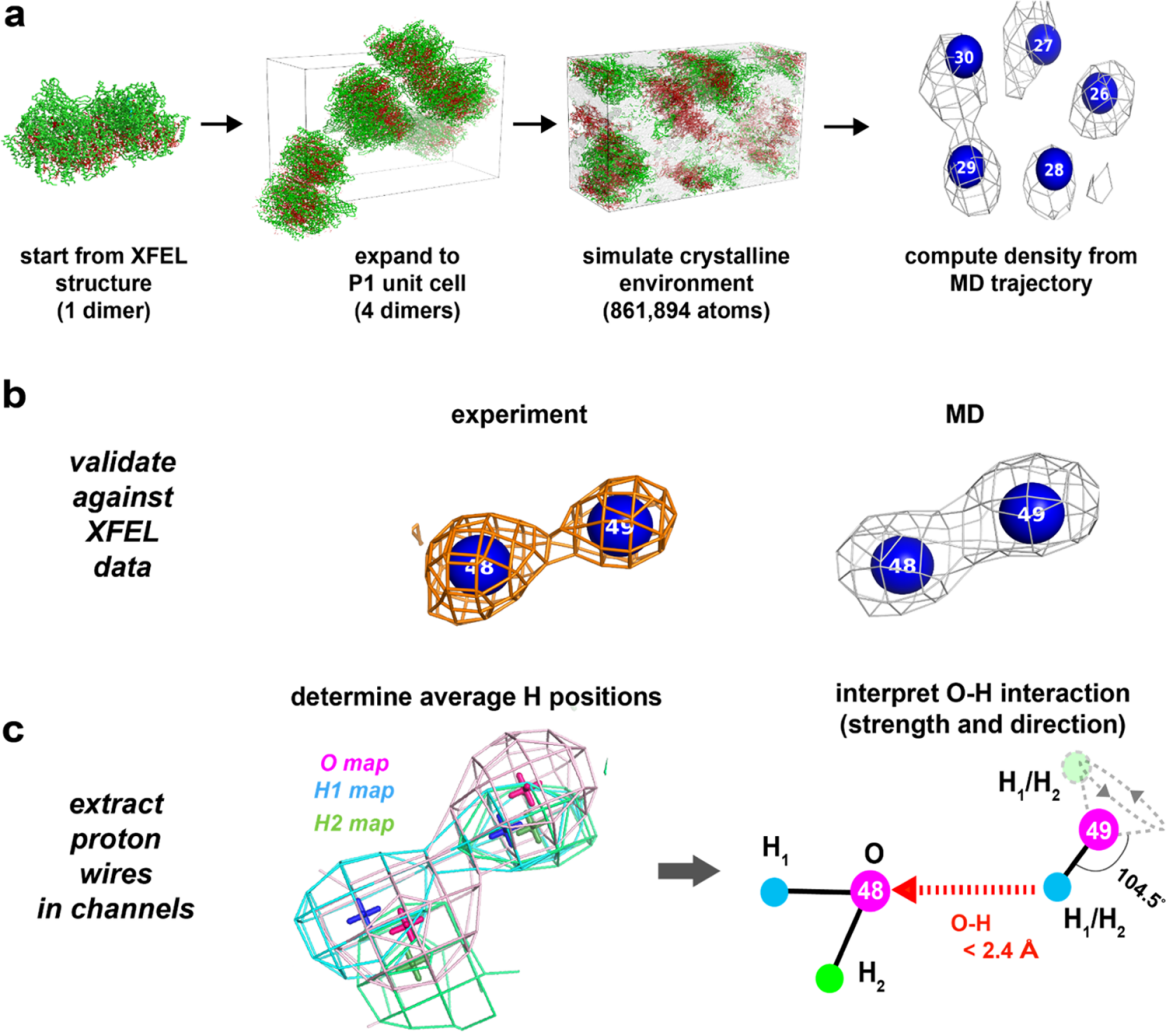
Methodological approaches used in this work. (a) The protein structure of PS II, which crystallizes in the P212121 space group, is first collected at an XFEL. The asymmetric unit is then expanded to the full P1 unit cell. Periodic boundary conditions are applied, wrapping the protein coordinates around the simulation box. The simulation is then carried out in a box approximately the size of the unit cell, with waters shown in light gray, protein atoms shown in green, and lipid/cofactor atoms shown in red. After carrying out the simulation, electron density maps are computed from the trajectory. (b) Comparison of experimental density (1σ/0.173 e/Å^3^) to the simulated water density (2.08 e/Å^3^) is first required to make sure the XFEL electron density is reproduced with high fidelity. (c) Once the validation step is complete, simulated oxygen (purple, shown at 2.08 e/Å^3^) and hydrogen (H1, cyan; H2, green, both shown at 0.26 e/Å^3^—see the Supporting Information) densities are computed for the hydrogen-bonding analysis. Average simulated oxygen and hydrogen peak positions, extracted from the densities, are marked as crosses. An example schematic, demonstrating our interpretation of the hydrogen and oxygen maps around W48 and W49, is shown on the far right. W48 and the H1 and H2 associated with it are likely fairly rigid over the course of the simulation, while the H1 and H2 associated with W49 are likely more mobile and are swapping into and out of the hydrogen-bonding position. The nonbonded hydrogen can sit anywhere on the dotted cone.

We developed an MD model for crystalline PS II in the dark resting S_1_ state. The model consists of a single periodic P1 unit cell with four copies of the PS II dimer (one of the four dimers is shown in Figure 1a). Our protocol, which was developed initially by Wall et al.,^46^ allows for a direct comparison of the electron density between the simulation and the experimental room-temperature XFEL crystallography data. The procedure that was followed for the analysis of the water and proton channels in PS II is summarized in Figure 2 and is briefly described here. The scattering contribution of each atom (including hydrogens) in the simulation box (i.e., unit cell) was superimposed to calculate an electron density map in the symmetry setting for the crystal structure (Figure 2a, and see Section 2 and the Supporting Information for details). The electron density calculation was done using multiple snapshots of atomic coordinates extracted from a range of simulation time thus yielding an average electron density map that can be compared directly with the electron density map of the XFEL room-temperature crystallography data. To yield a satisfactory visual comparison between MD and experimental maps, we show all MD maps in this study at a level of 2.08 e/Å^3^, unless explicitly stated otherwise (see Figure S1).

After an initial MD simulation that included a 45 ns equilibration run, a 55 ns production run was carried out for analysis. Mean structure factors and electron densities were computed from this trajectory and analyzed to investigate the water structure and hydrogen bonding networks in the channels surrounding the OEC. For simplicity, we focus our discussion of the analysis on one of the two monomers in the PS II dimer unit: the monomer referred to as “unlocked” (so-called because the subunits of this monomer have less contact with the neighboring PS II dimer in the crystal packing).^54^ This monomer corresponds to chains annotated as uppercase in the deposited PDB 7RF2.

To gain insight into the simulated water structure around the active site, an average water MD density map was computed over the last 55 ns of the trajectory and compared to the crystallographic-derived water electron densities. To obtain a detailed picture of the independent contribution of each water to the MD density, the isolated electron density maps for specific waters were also computed (Figure 2a,b). These maps allowed us to understand the origins of specific patterns in the simulated MD density and to gain insight into the mobility of the waters (see Figure S2). We start by validating the MD-derived water density (which we will refer to as the MD density) against the crystallographic waters from the crystal structure obtained from XFEL data (Figure 2b).

To analyze the strength and orientation of the hydrogen-bonding network from the simulations, we developed a novel analysis technique, called MADI—Map-based Acceptor–Donor Identification (see the Supporting Information). MADI analyzes MD hydrogen and oxygen maps to obtain information about mean simulated water positions and orientations. We note here that fixed point charge force fields like those used in this work lack higher-order electrostatics terms needed to precisely describe hydrogen bonding interactions. Nonetheless, the mean field approach in these force fields has been successful, on average, in reproducing basic geometric properties of hydrogen bonding that are relevant to our study.^55,56^ The information from MADI is then used to identify putative hydrogen bonds and to produce a measure of their strengths (an example is shown in Figure 2c). Uniquely, MADI also provides a means for explicit donor and acceptor identification. This information is interesting to examine in the context of the previously hypothesized changes in the directions of donor–acceptor pairs before and after proton transfer.^40^ See Table S5 for a complete list of significant water wires identified by MADI.

### 3.1 MD Density and Comparison with XFEL Experimental Data

**Figure 3:**
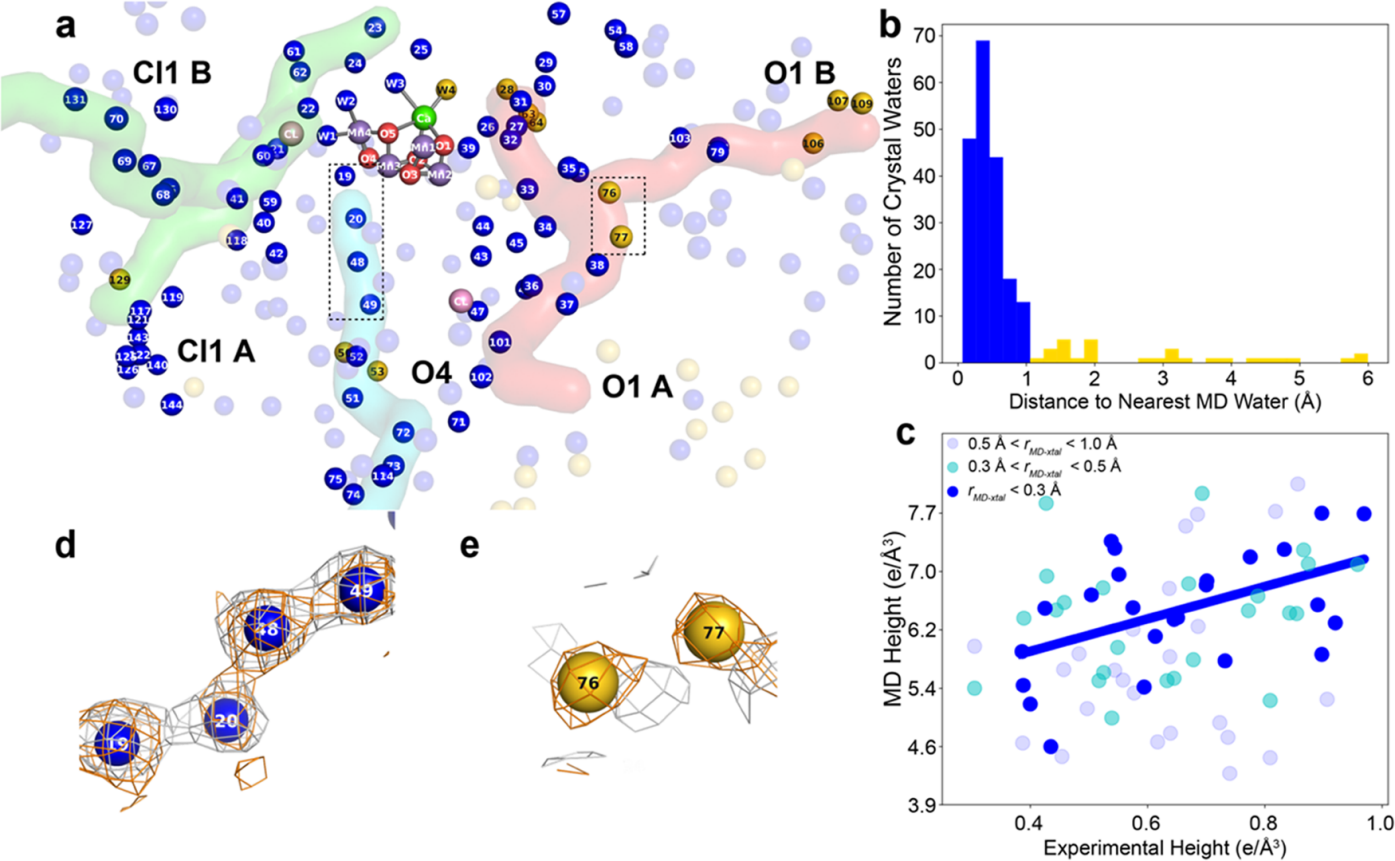
Comparing the MD-derived electron density for waters with the crystallographic electron density. (a) Crystallographically modeled waters around the OEC, color-coded by their relative agreement to the MD density. Blue-colored waters are less than 1.0 Å from a peak in the simulated electron density map, while gold-colored waters are more than 1.0 Å away from the nearest simulated peak. The three water channels of interest: The O1 channel (red), Cl channel (green), and O4 channel (blue) are also overlaid. (b) Distribution of distances between crystallographic water positions and MD water density peaks within a 25 Å radius from the OEC (centered on O1). (c) Scatterplot showing the correlation between the MD and experimental electron density peak heights for different groups of MD waters in the inner (blue), middle (cyan), or outer (light blue) shell. (d) Example of a region with close MD water peaks and corresponding crystallographic waters. The simulated MD water density (gray contour at 2.08 e/Å^3^) is shown in the context of the crystallographic waters (numbered blue spheres). The experimental 2Fo-Fc map is overlaid (orange) and contoured at 1σ. (e) Example of a region with distant MD water peaks and corresponding crystallographic waters. The simulated MD water density (gray and contoured at 2.08 e/Å^3^) is shown in the context of the crystallographic waters (numbered yellow spheres). Experimental 2F_o_–F_c_ map is overlaid (orange) and contoured at 1σ. The water numbering convention for all waters in the vicinity of the OEC has been described previously.^8^ The numbers of waters are based on the 0F (S_1_ state) experimental crystal structure used by Hussein et al.^8^ (PDB ID Code 7RF2). The channels were mapped by Caver 3.0 Pymol plugin using this room-temperature crystal structure.^57,58^

After calculating the MD density, we compared it to the crystallographic waters around the active site. In Figure 3a, the crystal waters surrounding the OEC are displayed along with the channels of interest. Out of the 222 crystallographic waters within a 25 Å radius from the OEC (centered on O1), 189 had a strong (>2.08 e/Å^3^) MD water density peak within 1 Å of the crystallographic water positions (see Figure S3 for a visual of this ROI in the context of the full PS II dimer as well as the monomer/monomer interfacial region). The agreement in all three channels was generally better toward the interior of the protein than toward the bulk. The O1 channel contains the most waters whose positions were not recovered by the simulation.

To compare the MD water density with the XFEL crystallographic data, we define *r*_MD-xtal_ as the distance between the crystallographic waters and the nearest MD water density peak. The histogram in Figure 3b shows the distribution of *r*_MD-xtal_. The average displacement is around 0.77 Å. Figure 3d,e illustrates how densities for both “close” (*r*_MD-xtal_ < 1 Å) and “distant” (*r*_MD-xtal_ > 1 Å) MD waters look in the context of the crystallographic waters and density; the visual agreement is consistent with the close vs distant classification.

We next analyzed the height of the peaks extracted from the simulated MD density. In Figure 3c, simulated peak heights are compared to the corresponding experimental peak heights. Their relationship was quantitatively analyzed by computing the Pearson correlation coefficient for three sets of MD water density peaks: those within the outer (0.5 Å < *r*_MD-xtal_ < 1 Å), middle (0.3 Å < *r*_MD-xtal_ < 0.5 Å) and inner (*r*_MD-xtal_ < 0.3 Å) shell about the corresponding crystallographic water. The Pearson correlation coefficient is −0.014 for the outer peaks, 0.210 for the middle peaks, and 0.496 for the inner peaks. Thus, on average, the closer an MD water peak is to its corresponding crystallographic water, the stronger the agreement between experimental and simulated peak heights.

We compared simulated peaks located in each of the channels to ∼1320 randomly selected peaks found at least 28 Å from the OEC. We differentiated between inside and outside of the channels by using amino acid residue pairs to define channel boundaries (see Tables S6–S8). Intrachannel waters exhibited greater overall peak height (mean peak height of 5.75 e/Å^3^) than those which were closer to the bulk (mean peak height of 3.85 e/Å^3^). Assuming a higher peak height corresponds to a lower mobility, this difference suggests that individual water positions within the channels are less variable in the simulation, allowing for density to build up to more elevated levels (see Figure S4).

As in previous crystalline MD studies where the protein is restrained to the crystal structure,^45,46^ the number of strong peaks in the MD water density map exceeds the number of crystallographic waters in our simulation. For example, there are 249 strong peaks in the MD density within a 20 Å radius of the OEC (coordinates available at https://github.com/mdoyle17/Molecular-Dynamics-Analysis-in-Python), compared to 161 waters in this region of the crystal structure. A recent unrestrained solution-state MD study by Sirohiwal and Pantazis^59^ of a single PS II monomer found 182 MD waters in this same region, using a different analysis method based on counting waters close to the protein every 105 ps. The increased number of peaks in our simulation compared to that of Sirohiwal and Pantazis is consistent with our previous finding that protein restraints can increase the number of strong peaks in the water density.^46^ It is important to note that, although crystallographic waters can be matched to equivalent waters in unrestrained MD simulations by identifying the sites on the protein where they interact,^60^ at present restraints are necessary for accurately recovering the positions of ordered waters using density maps, and that increasing the agreement when restraints are relaxed is a key strategy for improving MD force fields in the crystalline phase.^46^

Using the simulated solvent map as a guide, we assessed the potential of using the MD water peaks to improve the water model in the crystal structure, similar to what was done in a previous MD study.^45^ After remodeling six new waters into the deposited 0F (S_1_ state) structure (PDB ID 7RF2) based on peaks found in the MD solvent map, we observed visible improvement in the experimental 2F_o_–F_c_ map. Two examples are shown, involving newly solved waters W202 (occupancy of 0.59 and *B*-factor of 29.01) and W205 (occupancy of 0.72 and *B*-factor of 36.94) that only register subsequent to MD simulations (Figure S5). These results also show that there is scope for using the MD simulations to model additional waters with much weaker electron density within a 20 Å radius of the OEC. The six waters mentioned above were not added to our deposited XFEL model (PDB ID 7RF2) by the automatic water picking algorithm in the structural refinement software based on default threshold parameters. However, when they are manually modeled in and refined, there is enough electron density for these waters to be retained after filtering during automated refinement. Thus, our crystallographic data support the addition of six waters within a 20 Å radius of the OEC that were purely suggested by the MD simulations. This finding is consistent with a recent study reporting that MD simulations can be used to enhance crystal structures by locating additional ordered waters that are supported by the crystallographic data.^45^

Next, we transition from a broad description of the MD density in the region surrounding the active site toward a more in-depth, channel-specific analysis. By using results from our simulated MD-based electron density maps, we can explore water mobility and gain insight into hydrogen bonding networks within the O4, O1, and Cl1 channels.

### 3.2 O4 Channel

**Figure 4:**
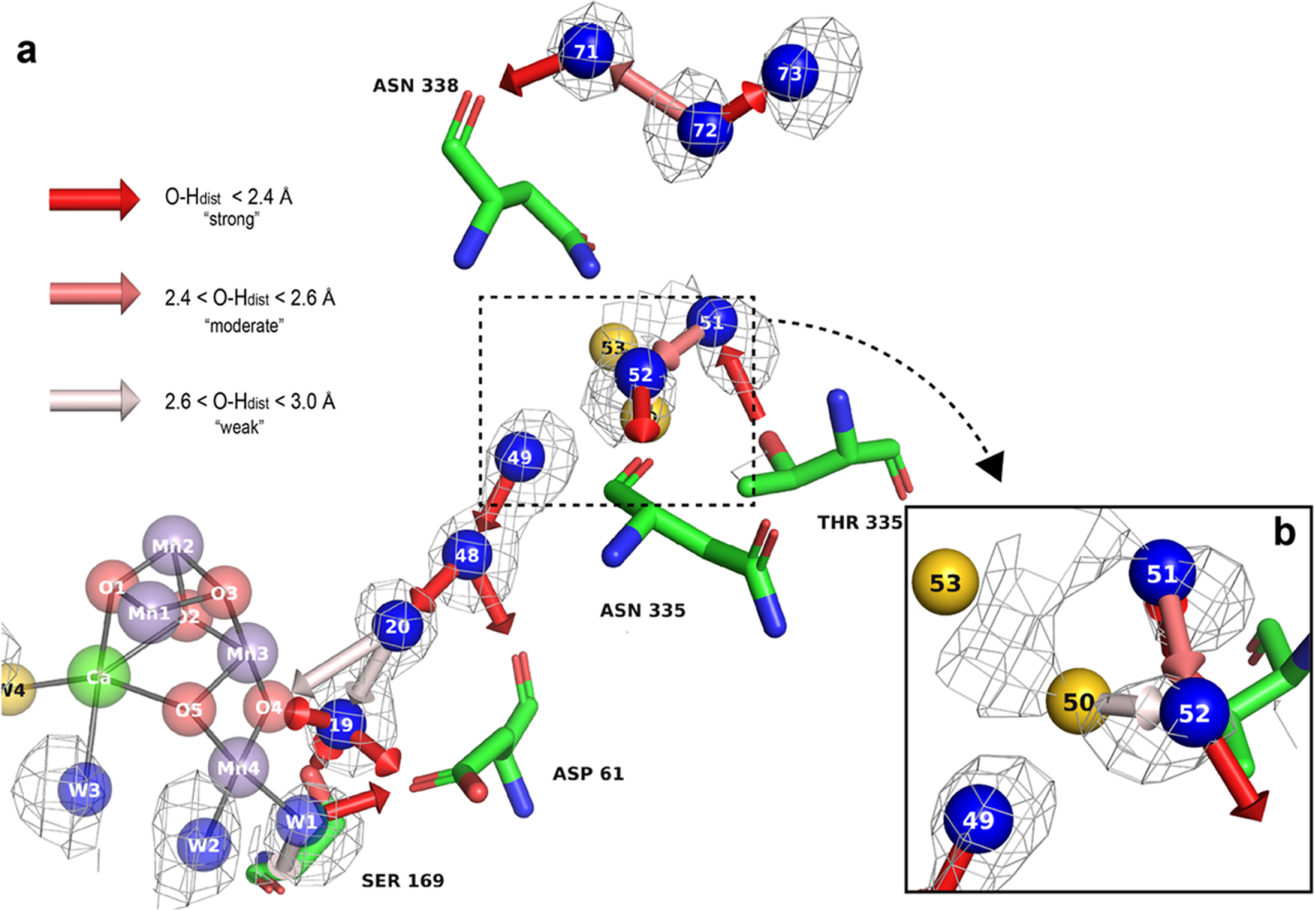
Hydrogen-bond and water dynamics in the O4 channel. (a) Simulated MD solvent map in the O4 channel (gray, contoured at 2.08 e/Å^3^) along with identified hydrogen bonds, overlaid with the crystal structure (numbered, in color). The results from the hydrogen bonding analysis are depicted as arrows, pointing from the donor toward the acceptor. The strength of the bond identified by our algorithm is represented by a color gradient as defined in the legend. (b) Rotated view of the W50–W51–W52–W53 region, showing that crystallographic waters W50 and W53 lie outside of the MD map wireframe. All simulated maps were carved at a 2 Å radius about each atom. Oxygen and hydrogen peaks from the MADI analysis, demonstrating the average orientation of waters showing favorable hydrogen-bonding geometries in this channel, can be found in Figure S8.

The O4 channel, a proposed proton channel, contains one main donor–acceptor chain, which splits off at several points, as revealed by the MADI analysis (Figure 4a). The chain originates at W49 and proceeds toward the active site through W48–W20–W19, branching toward O4 (O4 is a bridging ligand in the Mn_4_CaO_5_ cluster, and the reason for the O4 channel nomenclature) at both W20 and W19, and also branching off toward ASP 61 at W48 and W19. The simulated waters in the neighborhood of the Mn_4_CaO_5_ active site more closely reproduce the crystallographic water positions, and have higher peak heights, relative to the waters farther up the channel.

A dense cluster of seven strong bonds exists close to the OEC. This has the potential to create kinetic barriers to water mobility, leading to a buildup of density. This is demonstrated by the high average simulated water peak height of 6.79 e/Å^3^ in the W19–W20–W48–W49 region (see Figure S6). This high value (relative to the average peak height value of 6.01 e/Å^3^ associated with the 222 waters within a 25 Å radius of the active site, centered on O1), paired with the excellent agreement between the MD water density peaks and crystallographic water positions for this quartet of waters (mean *r*_MD-xtal_ of 0.22 Å), suggests that the structure of these waters is quite rigid.

Moving toward the W50–W51–W52–W53 region (Figure 4b), the simulated water density weakens (mean MD water density peak height of 4.62 e/Å^3^) and a lower overlap between MD and crystallographic densities is observed (average *r*_MD-xtal_ value of 1.31 Å). This supports the idea that there is (1) higher mobility and (2) greater conformational diversity sampled by the simulated waters in this region. The lack of an H-bond connection to the rest of the main chain suggests that MD waters are moving more freely in this zone (see Figure S7).

**Figure 5:**
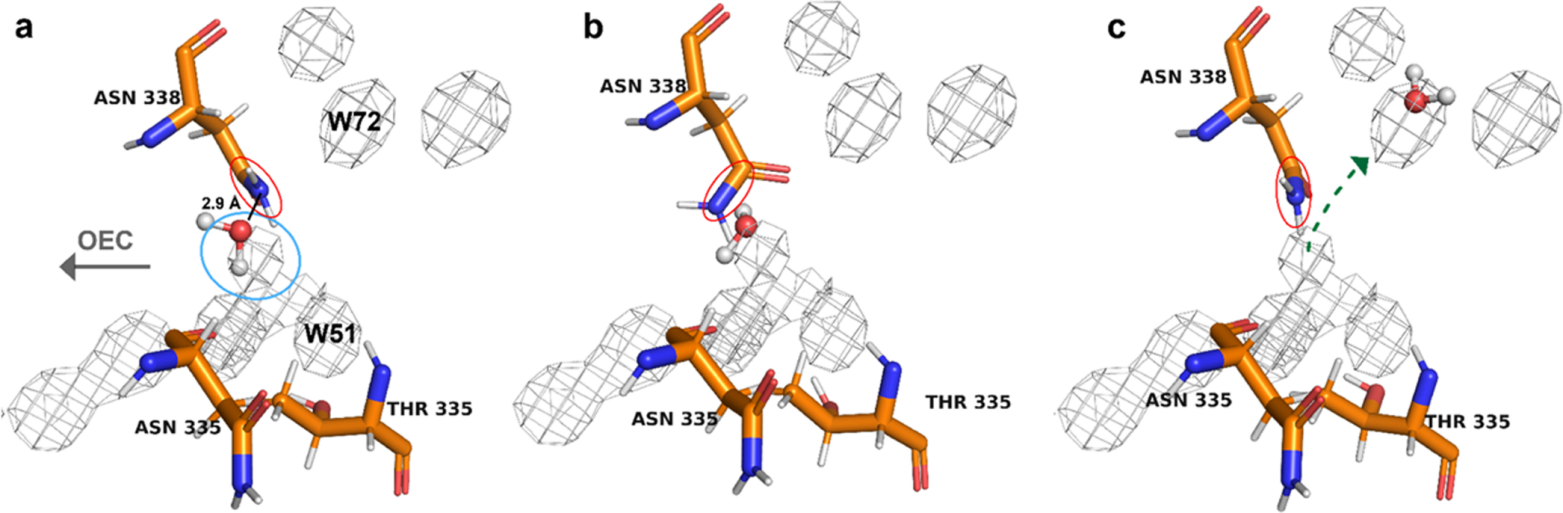
Drawbridge mechanism of ASN 338. Snapshots of the ASN structure from a single copy at (a) 90.94 ns, (b) 92.42 ns, and (c) 92.88 ns suggest that the ASN side-chain movement is coupled with water transfer away from the OEC. The average solvent map from the entire trajectory across all four copies is overlaid and contoured at 2.08 e/Å^3^. As the water molecule approaches ASN 338, it sits in the excess density, not found in the experimental map (blue circle). The side-chain momentarily flips, potentially providing more room for the water molecule to hop over. After the water molecule crosses over and fills the W72 position, the drawbridge raises. It is observed to continue to raise and lower for the rest of the simulation.

Snapshots from the MD trajectory reveal rare instances when the gap between W51 and W72 may be transiently bridged (see Figure S9). Immediately before/after a molecule passes through this gap, however, we observed waters located in a commonly sampled position nearby W51 and ASN 338 (Figure 5). We hypothesize that this position is frequently occupied by waters transiting this crevice, as the buildup of additional simulated water density here is visible at 2.08 e/Å^3^.

The movement of waters across this gap appears to be correlated with the rotation of the nearby ASN 338 side chain. A solvent molecule was observed moving from the aforementioned excess density into the W72 position during the last 10 ns of the simulation after the side chain of ASN 338 rotates ∼90°, resembling the “lowering of a drawbridge” (Figure 5a–c). We postulate that this provides more room for a molecule to hop over. A similar phenomenon, involving a Na^+^ ion, was also observed (see Figure S10). However, in our Cl^–^ simulation, where Cl^–^ ions likely screened charged areas within the channels and reduce the long-range electrostatic effects, no Na^+^ ions were observed approaching the OEC via the O4 channel.

### 3.3 O1 Channel

**Figure 6:**
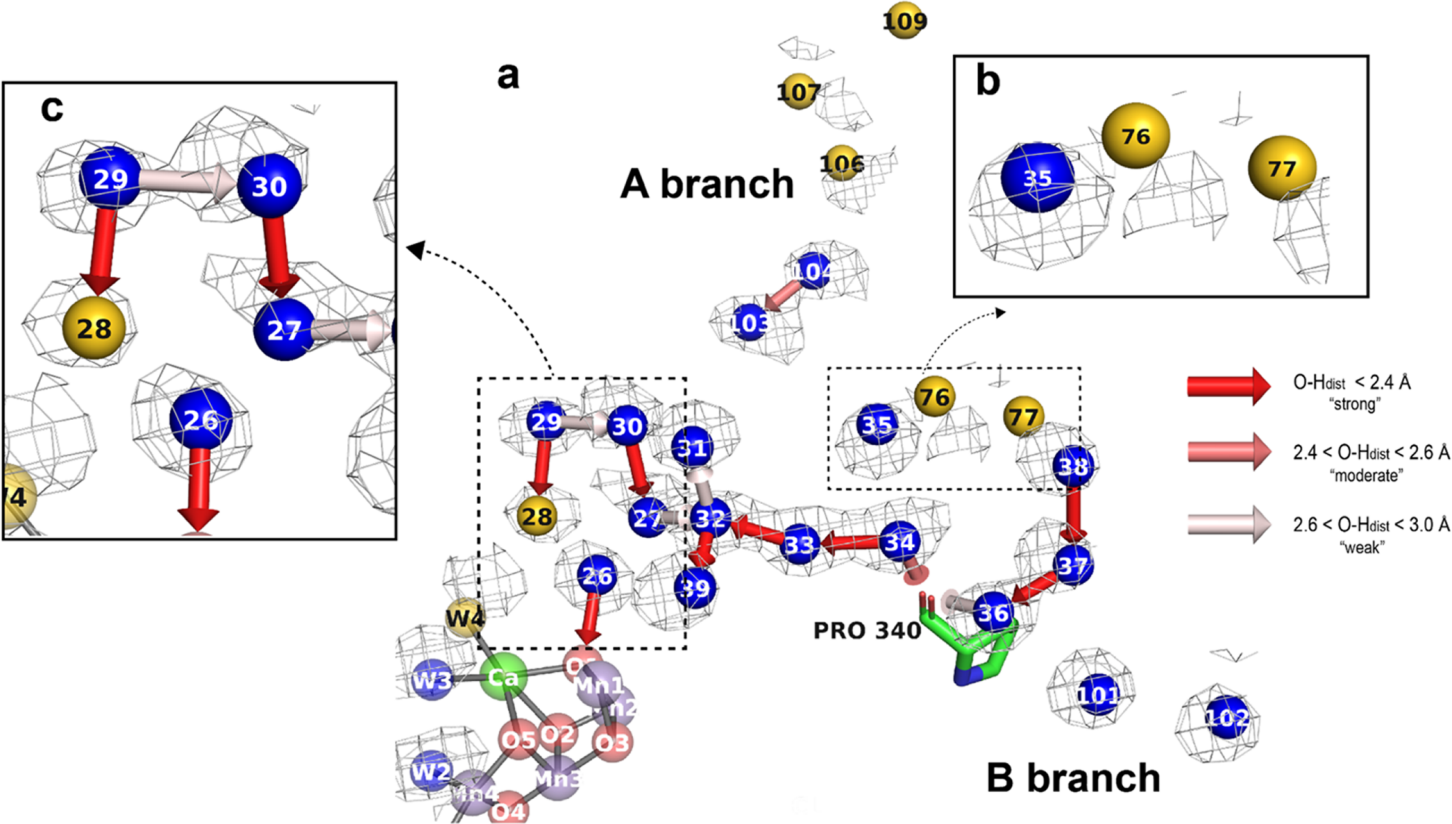
Hydrogen bond and water dynamics in the O1 channel. (a) Simulated MD solvent map in the O1 channel (contoured at 2.08 e/Å^3^) along with identified hydrogen bonds, overlaid with the crystal structure. The results from the hydrogen-bonding analysis are depicted as arrows, pointing from donor toward acceptor. The strength of the bond identified by our algorithm is represented by a color gradient. (b) W76 and W77, where there is significant disagreement between the map and crystal structure. (c) Crystal water and MD map agreement/H-bond network in the region of the penta-cluster waters (W26–W27–W28–W29–W30). All simulated maps were carved in a 2 Å radius about each atom. Oxygen and hydrogen peaks from the MADI analysis, demonstrating the average orientation of waters showing favorable hydrogen-bonding geometries in this channel, can be found in Figure S11.

Several consecutive, short, donor–acceptor chains were identified by the MADI analysis in the O1 channel, a proposed water channel (O1 is a bridging ligand in the Mn_4_CaO_5_ cluster, and the reason for the O1 channel nomenclature) (Figure 6a). They were found in both the penta-cluster of waters (also referred to as the water wheel^7^) by the Mn_4_CaO_5_ active site (W26–W27–W28–W29–W30) (Figure 6c), and around the main channel trunk (W31–W32–W39–W33–W34), although no chain was found directly connecting this channel to the OEC. Toward the ends of branches A and B, no chains were identified. The simulated waters in the trunk of the channel were found to have strong peak heights and showed overall better agreement with crystallographic waters relative to those closest to the active site and in the channel branches.

The closest chain to the active site was found to originate in the penta-cluster at W29, passing through W30–W27–W32 before splitting off between W31 and W39. The average *r*_MD-xtal_ value of 0.61 Å in the penta-cluster is nearly 3 times larger than what was found for the waters closest to the active site in the O4 channel. Moreover, the O1 waters close to the active site have, on average, comparatively smaller peak heights (5.83 e/Å^3^) than those closest to the active site in the O4 channel (6.79 e/Å^3^), suggesting that the O1 channel waters are less rigid over the course of the simulation.

Within the penta-cluster, the individually calculated densities for W27 and W28 were the weakest, suggesting that they correspond to more readily exchangeable positions (see Figure S12). Additionally, both W28 and the nearby W4 are, on average, offset from the crystallographic positions by at least 1 Å. This is especially notable as W4 was moderately restrained to its initial position like the other three waters ligated to the Mn_4_CaO_5_ cluster (see Section 2). Therefore, its movement, despite these applied restraints, indicates that the model in this region should be a focus of future optimization of the MD simulation.

As one moves into the trunk of the channel, the average *r*_MD-xtal_ value improves to 0.40 Å, and the mean MD water peak height increases to 6.40 e/Å^3^. However, individual water density analysis in this region indicates that these waters undergo fast and well-coordinated movements (with water exchange occurring on <15 ns timescale), while simultaneously preserving well-defined average positions (see Figure S2).

Toward the bulk water region, the average *r*_MD-xtal_ value decreases and the simulated water density weakens in most areas. The largest deviation is for W76 and W77 (Figure 6b) which are on average 1.88 Å away from the crystallographically modeled positions, and exhibit a weak average peak height of 4.84 e/Å^3^. Individually calculated water maps (Figure S13) indicate that mobility is relatively high in this zone, which might contribute to the decreased agreement with the crystal structure. MD waters toward the end of branch A also demonstrate greater average offset from the corresponding crystal positions (average *r*_MD-xtal_ value is 1.0 Å) relative to the trunk region. They also correspond to a weak average peak height (5.19 e/Å^3^), with no significant nearby donor–acceptor chains.

### 3.4 Cl1 Channel

**Figure 7:**
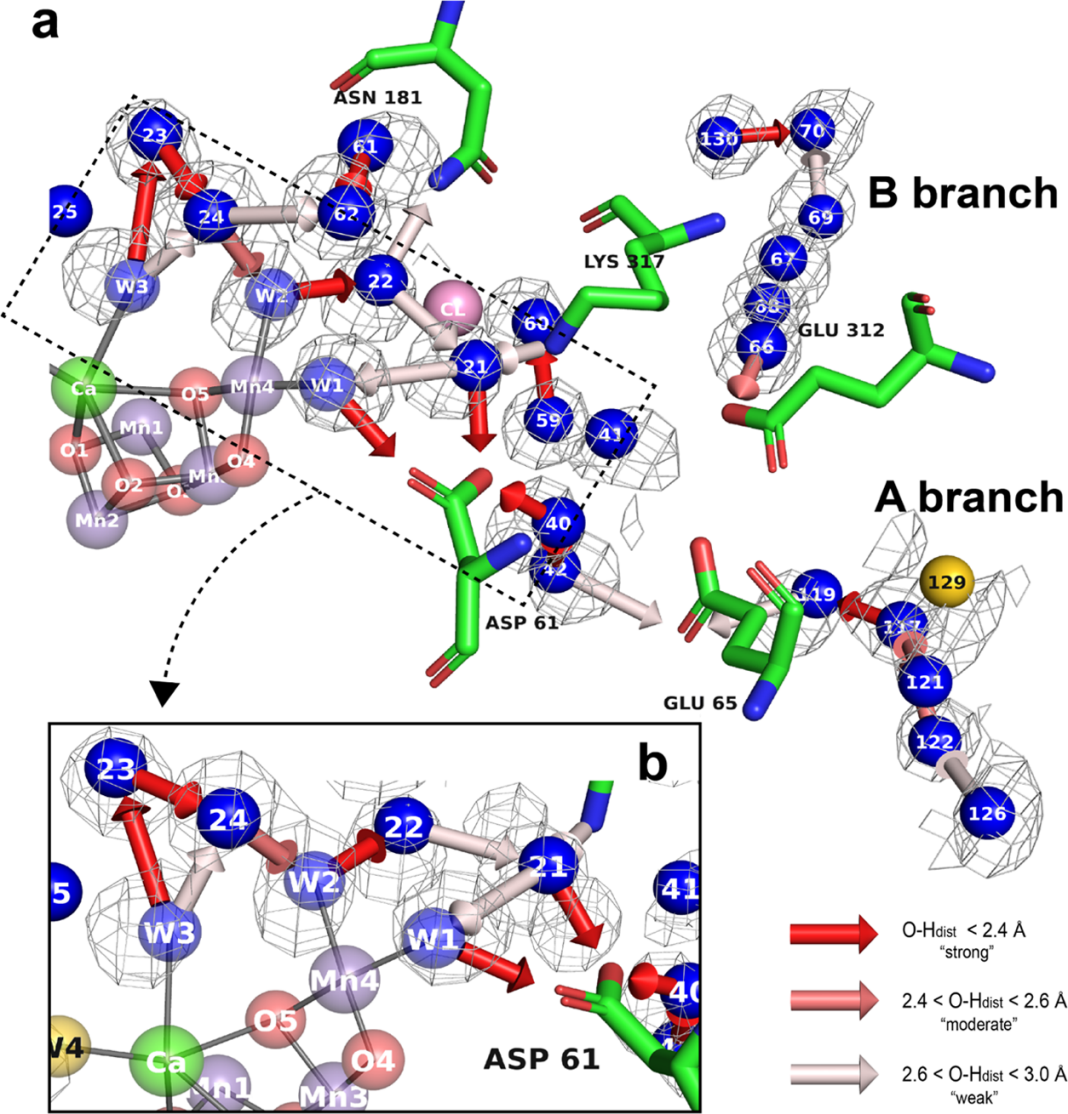
Hydrogen bond and water dynamics in the Cl1 channel. (a) Simulated MD solvent map in the Cl1 channel (contoured at 2.08 e/Å^3^) along with identified hydrogen bonds, overlaid with the crystal structure. The results from the hydrogen-bonding analysis are depicted as arrows, pointing from donor toward acceptor. The strength of the bond identified by our algorithm is represented by a color gradient. (b) Continuous hydrogen bond wire from W3 to ASP 61, identified through our MADI analysis. Only the core participating waters are shown. Peaks found from MADI analysis in this channel can be seen in Figure S15.

A long, unidirectional hydrogen-bonded chain identified via MADI analysis was found in the Cl1 channel (Figure 7a), a proposed proton channel in the S_2_ to S_3_ transition,^8,43,61^ originating from W3 and pointing away from the OEC via W23–W24–W2–W22 before forking toward ASN 181 and W21. From W21, it splits toward W1 and ASP 61 (Figure 7b). Overall, the Cl1 channel shows the best agreement between MD water density peaks and crystallographic waters (see Figure S14). MD waters in branch B demonstrated especially high agreement with the crystal structure and contained strong peaks.

We postulate that the large number of nearby side chains, as well as anchoring effects provided by the participating ligand waters (W1, W2, and W3) in the OEC, play a role in directing the long hydrogen-bonded chain described above. As one moves further along the chain through W40–W41–W42, the average *r*_MD-xtal_ value remains close (0.65 Å) and the average peak height remains high (6.24 e/Å^3^). The MADI H-bond directions do not form a unidirectional chain in this region, therefore no clear picture is provided regarding proton transfer in the S_1_ state structure we used in this study. We note, however, that a connection is still forged through ASP 61 and GLU 65 out toward the end of branch A. In our recent XFEL crystallography study of the S_2_ to S_3_ transition, it has been observed that this network appears to be poised to realign, via a rearrangement of the GLU 65/GLU 312 bottleneck region (proposed as a proton gate), enabling transfer of the proton out to the bulk via GLU 65, likely connected to a water deprotonation step upon a substrate water insertion at the OEC.^8^

In the current MD model, the lack of a shared proton modeled between GLU 65 and GLU 312 relaxed the bottleneck so that its width at the final snapshot of the trajectory was >3 Å across all four PS II dimer copies. Since the protonation state of residues is expected to influence water movement, studying the effects of charge sharing between GLU 65/GLU 312 on hydration in the Cl1 channel will be a focus of future work. With our current model, additional locations with elevated water density in the MD density were observed both inside and outside this GLU 65–GLU 312 “proton gate” (see Figure S16). Other species such as Na^+^ ions were also observed moving toward this bottleneck region—likely drawn in by the negatively charged environment. However, no Na^+^ ions were found moving into this negatively charged pocket from the Cl^–^ simulation. This difference might be attributed to alterations in the charge distribution surrounding the channel through adding additional Na^+^ and Cl^–^ ions.

As one moves toward the bulk water region, a characteristic difference in mobility between branches is observed. Specifically, waters toward the end of branch B have very high agreement with the MD density (average *r*_MD-xtal_ value of 0.30 Å) and a strong average peak height of 6.61 e/Å^3^. In branch A, however, the average *r*_MD-xtal_ value is 0.73 Å and the average peak height is only 5.54 e/Å^3^. The trend between these two branches also aligns with the previous experimental *B*-factor analysis.^8^

## 4 Discussion

Our study used calculated electron density maps computed from room-temperature crystalline MD simulations to interpret the water dynamics of PS II and infer hydrogen-bonding networks across three channels of interest in the dark resting S_1_ state, obtained from the room-temperature serial femtosecond crystallography data measured at an XFEL. Building on a previous MD study of water structure in endoglucanase,^46^ we applied the crystalline MD method to PS II, which is a substantially larger system (nearly 6 times as many atoms as the cited study).

**Figure 8:**
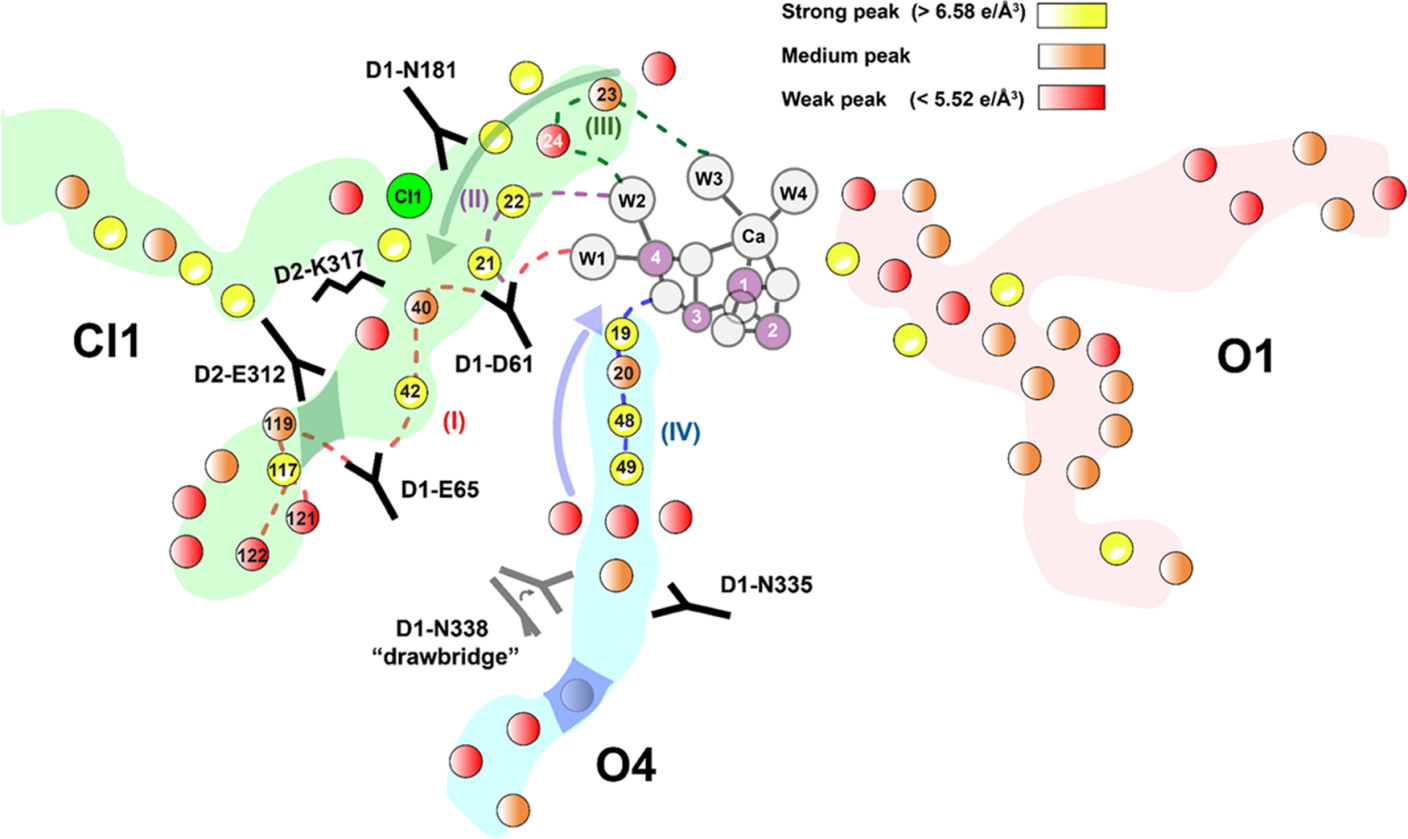
Overview schematic of potential proton pathways, with arrows showing the direction of the hydrogen-bond networks inferred for the S_1_ state, along with highly dynamic regions in each of the three channels. We are interpreting the low-density peaks (in the 33rd percentile for waters within a 25 Å radius of the active site, centered on O1) as corresponding to more mobile waters, and the high-density peaks (above the 66th percentile for waters within a 25 Å radius of the active site, centered on O1) as corresponding to more rigid waters. Two previously discussed bottleneck regions in the Cl1 and O4 channels are shaded. Possible proton pathways from the OEC to the bulk are indicated by distinctly colored dashed lines, labeled I–IV. Selected waters which are thought to participate in each pathway are labeled based on the numbering convention described previously.^8^

We achieved a high level of reproducibility of crystallographic waters; within a 25 Å radius of the OEC (centered on O1), ∼85% of the crystallographic waters were recovered within 1 Å of their crystallographic position. There is a trend for the MD waters to have higher electron density in the channel regions compared to waters in the bulk, which indicates that the water structure is more ordered within the channels (see Figure S4). We discuss below the relevance of our findings in the context of the function of the enzyme. Figure 8 summarizes our primary findings from this work to accompany the discussion.

### 4.1 Networks for Proton Transfer

Given the fact that we are reporting on results derived from a classical MD simulation, we are unable to capture the quantum mechanical aspects of the proton transfer process. The intricacies involved in analyzing proton transport in biological systems, and specifically the tunneling processes that can govern such transport, have been described elsewhere.^62−64^ However, this current work is simply focused on the dark resting S_1_ state of PS II with no reaction activated. We thus conjecture about the proton transfer pathways used during the reaction cycle based on hydrogen-bond network orientation and connectivity inferred in the S_1_ state.

Our MADI analysis provides us with information regarding the strength and directionality of the inferred hydrogen bond network. This information provides a way to consider the potential proton release pathways through the channels. We note that our results do not provide supporting evidence for a proton transfer pathway in the O1 channel. Unlike in the O4 and Cl1 channels, MADI did not reveal a significant hydrogen bond wire in the O1 channel which is connected to the cluster. Furthermore, as one moves out toward the ends of branches A and B of the O1 channel, very few hydrogen bonds are detected, making the potential path for proton transfer significantly unlikely. Disruption of the network in the O1 channel was also seen by Kaur et al. in a study which combined MD with Multi Conformation Continuum Electrostatics (MCCE) and Network Analysis. In their work, such disruption was observed 10 Å away from the OEC, and due to the high level of interconnectedness found around the active site, a released proton had potential to access all three channels. However, the O1 channel was not as likely to be the proton release path.^65^

In the case of the O4 channel, we observe that the donor-to-acceptor direction is from the bulk to the OEC, similar to a post-proton transfer orientation discussed by Sakashita et al.^40^ It has been proposed from theoretical studies that the O4 channel is likely used for a proton release during the S_0_ → S_1_ transition,^66,67^ which is supported by the data presented here as it is from the S_1_ state. For the Cl1 channel, the donor-to-acceptor orientations are mostly from the OEC to the bulk, a pre-proton transfer orientation, which indicates the Cl1 channel is primed to release a proton upon a deprotonation event in a subsequent transition beyond the S_1_ state. This channel has been proposed previously as a proton release pathway during the S_2_ → S_3_ transition based on p*K*a calculations of residues, FTIR spectroscopic studies, and more recently in MCCE simulations,^43,61,65^ as well as in the snapshots of room-temperature crystallography data taken during the S_2_ → S_3_ transition.^8^ In contrast to previous computational studies of the hydrogen bond network primarily in the O4 channel, our MADI analysis is able to determine the strength and direction of the inferred network in all of the channels.

For the Cl1 channel, we catalog multiple plausible pathways depending on the origin of the proton (W1, W2, or W3). A proton originating at W1 would be transferred to ASP 61 and donated to W21 or W40 after a flip in the H-bond orientation of ASP 61. We note that W21–LYS 317 is a dead end, so the proton is most likely to travel through W40–W42–GLU 65 (path I in Figure 8). At this point, it can continue out toward the bulk via branch A.

A proton originating at W2 could move along W22–W21–ASP 61 (path II in Figure 8) before carrying on the same route as a W1 proton (we note that the W2–W22–ASN 181 pathway is a dead end). In the case of a proton starting at W3, the pathway would primarily use the W2 route after getting shuttled through W23 and W24 (path III in Figure 8). A recent paper by Allgöwer et al. suggests path III to be a strong candidate for proton transfer in the S_2_ → S_3_ transition based on multiscale quantum and classical simulations.^68^

Based on the MADI analysis (see Figure 4), a proton released from the O4 channel would likely originate from the O4 atom of the OEC before traveling up the quartet of rigidly fixed waters W19–W20–W48–W49 (see Figure S6) via a Grotthuss mechanism, which involves a free proton hopping from one oxygen to the next. The next steps are less clear and require the formation of multiple transient bonds in the mobile W50–W51–W52–W53 region, as well as between W51 and W72, before a proton can advance farther up the channel. The latter likely involves movement of ASN 338 as seen in our simulations (path IV in Figure 8).

### 4.2 Water Transport

Previous steered MD simulations carried out to study the energetics of water permeation in PS II have revealed that no channel permits absolutely unrestricted access of water to the OEC.^37^ The idea that water transport is a controlled process because of the very oxidative potentials present at the catalytic Mn site, and which involves regulatory mechanisms to prevent bulk water from being present at the active site, makes studying water dynamics within the channels important.

In the present simulation, we found that waters in the Cl1 channel exhibited the best all-around agreement with the crystallographic waters. We postulate that the Cl1 channel is relatively rigid (see Figure S14). For water transport to occur in a given channel, we hypothesize that it is necessary, at the very least, for the region directly adjacent to the active site to be favorable for water mobility. According to this hypothesis, the likelihood of water transport in the O4 channel would be low due to the four rigid waters (W19–W20–W48–W49, see Figure S6) close to the active site.

Close to the OEC, the simulated water peaks in the O1 channel were, on average, weaker than those in the O4 and Cl channels. The O1 channel is also unique in the sense that it is the only channel that contains a crystallographic water near the OEC that is distant from the nearest MD water density peak (W28). Dynamic pockets in this channel are also observed toward the end of branch A with W106, W107, and W109, and in the middle of the channel near W76 and W77.

By analyzing individually calculated water maps, we also observed that quick exchange can occur in the O1 channel trunk (see Figure S2) even though average MD water positions in this region are well preserved. From this observation, we conjecture that some waters are capable of simultaneously preserving a well-defined average structure while exchanging through what are likely fast and well-coordinated movements. It will be interesting to further study the connection between the strength of the MD water density and the frequency with which waters occupying a site are exchanged during the simulation.

The aforementioned observations support the idea that the O1 channel is dynamic in multiple regions throughout, making this channel a top candidate for the delivery of substrate water. This is consistent with our recent XFEL crystallography data which, based on time point snapshots during the S_2_ to S_3_ transition, suggested that the O1 channel waters are highly mobile, making it a strong candidate for facilitating water transport.^7,8^ Additionally, the O1 channel has been found to be highly accessible to bulk waters through a simulation-based study carried out by Sakashita et al.^44^

The number of strong peaks in the MD water density exceeds the number of crystallographic waters as described earlier (see Section 3). This is expected, as in the experimental crystal structure weakly interacting and highly mobile waters may not be resolved due to the reduced occupancy of atoms in a given position. This is more so at room temperature than at cryogenic temperature because of the difference in mobility. The additional MD water density peaks can potentially be used to increase the number of waters modeled in the crystal structure, as was found in a study of cAMP-dependent protein kinase.^45^ Indeed, we found some isolated cases where the MD suggested reasonable additions of water molecules that were supported by the experimental data (see Section 3 and Figure S5). Sirohiwal and Pantazis also recently noted that the number of water molecules associated with PS II appeared to be higher in their MD simulation than in many PS II crystal structures.^4^ While the authors concluded that the decreased number of waters is due to the dehydration of the crystals, the absence of water in a crystal structure does not imply that a region is dry since larger voids are to be interpreted as being filled with bulk solvent. In addition, the number of waters observed in the previous crystal structures^4,7,8,32,69−71^ also differs due to the resolution and measurement temperature (cryogenic vs room temperature). Moreover, decreasing the detergent and water content in PS II crystals is a specific strategy that has been used to increase the resolution of the diffraction data,^72,73^ which tends to increase—not decrease—the number of ordered waters in the crystal structure, as shown in Table 1 by Sirohiwal and Pantazis.^59^ We also note that the S-state advancement shown in the XFEL data by Kern et al. proves there is no sample dehydration that would inactivate PS II and release Mn(II).^4^ Our findings therefore do not support the claim that the crystal structures are dehydrated compared to the MD simulations.

## 5 Conclusions

The results presented here have demonstrated how a crystalline MD simulation approach can be used to understand the role of water networks in the PS II enzyme. By calculating average electron density maps from the trajectory, we can compare experiment to simulation and identify potential mechanisms of water transport and networks for proton transfer. Our study also introduced the MADI approach for analysis of MD-simulated electron density maps to identify potential water hydrogen bonding networks. The result indicates that the O4/Cl1 channel has multiple hydrogen bond wires emanating from the OEC toward the bulk but which need to be bridged in certain sections by the motion of waters/amino acids. We also found in the O1 channel that the inferred hydrogen bond network is disconnected from the bulk and instead contains several mobile regions, including regions where fast exchange occurs beneath a well-structured average density. Thus, we postulate that the O4/Cl1 channels are most likely involved in proton transfer, with the Cl1 channel demonstrating the clearest path for a proton to travel out toward the bulk, while the O1 channel is likely involved in substrate water delivery.

While not a focus of the current study, crystalline MD also has the potential to resolve inadequacies in crystallographic modeling.^45^ Better modeling of the solvent, accounting for differences in symmetry-related copies and sampling of multiple conformations of mobile waters/amino acids are some of the improvements we can potentially make with crystalline MD.

Future MD investigations beyond the dark-stable S_1_ state presented in this work and into the meta-stable S-states as well as time points in between would shed light on the functioning of these channels during specific transitions. This will require reparameterizations of the Mn cluster, using quantum mechanical calculations of electronic structures that differ from the S_1_ state studied here. Obtaining protonation states of critical amino acids from neutron diffraction studies is another approach to further improve the MD model.^46^

## Data Availability

All relevant python scripts and notebooks have been uploaded to Github with additional details (https://github.com/mdoyle17/Molecular-Dynamics-Analysis-in-Python). A PDB coordinate file with all identified strong water peak positions from the MD simulation is also available on the same Github page. Other simulation-relevant files are available in the following publicly accessible folder: https://drive.google.com/drive/folders/1KueRlSRi8KB-p0pw7UDAgxGLlkBvkVA8?usp=sharing.

## Supporting Information Available

Additional description of methodology, including details related to the mean structure factor calculations, comparison of the MD map to the experimental map, and MADI analysis used in this work; MD to experimental map visual comparison (Figure S1); individual water analysis (Figures S2, S6, S7, S12, and S13); visualization of the chosen ROI (Figure S3); histogram comparing solvent peak heights inside vs outside the channels (Figure S4); MD-aided refinement example (Figure S5); oxygen and hydrogen peaks from the MADI analysis (Figures S8, S11, and S15); snapshots of ASN side-chain-related phenomena (Figures S9 and S10); distribution of distances between experimental and MD water positions (Figure S14); example of additional MD solvent density observed in Cl1 channel (Figure S16); schematic related to determining optimal O–H bonds (Figure S17); distribution of O–O and O–H distances retrieved from MADI analysis (Figure S18); MADI analysis-related flowchart (Figure S19); breakdown of lipids/cofactors/ions/atoms in the simulations (Tables S1 and S2); documentation of lipid/cofactor/ion preparation (Table S3); lipid/cofactor/ion charge information (Table S4); MADI-identified water wires (Table S5); information regarding the amino acids which define each of the channels (Tables S6–S8); and information regarding the number of MD peaks and crystallographic waters found in each channel (Table S9) (PDF)

## Notes

Los Alamos National Laboratory Technical Release LA-UR-22-31349
