# Supplementary material for: Water Networks in Photosystem II Using Crystalline Molecular Dynamics Simulations and Room-Temperature XFEL Serial Crystallography
Source: J Am Chem Soc. 2023 Jun 27;145(27):14621–35. doi: 10.1021/jacs.3c01412 (PMC10347547; doi:10.1021/jacs.3c01412)
Supplement: Supplementary file 1 — ja3c01412_si_001.pdf [file ja3c01412_si_001.pdf]

Water Networks in Photosystem II using Crystalline Molecular-Dynamics Simulations and Room-Temperature XFEL Serial Crystallography

Margaret D. Doyle[1, †], Asmit Bhowmick[1, †], David C. Wych[2,3], Louise Lassalle[1], Philipp S. Simon[1], James Holton[1,4,5], Nicholas K. Sauter[1], Vittal K. Yachandra[1], Jan F. Kern[1], Junko Yano[1,*], Michael E. Wall[2,*]

[1]Molecular Biophysics and Integrated Bioimaging Division, Lawrence Berkeley National Laboratory, Berkeley, CA 94720, USA

[2]Computer, Computational and Statistical Sciences Division, Los Alamos National Laboratory, Los Alamos, NM, 87545, USA

[3]Center for Non-linear Studies, Los Alamos National Laboratory, Los Alamos, NM, 87545, USA

[4]Department of Biochemistry and Biophysics, University of California, San Francisco, San Francisco, California, CA 94158, USA

[5]SSRL, SLAC National Accelerator Laboratory, Menlo Park, CA 94025, USA

†Co-first authors with equal contribution

[*]To whom correspondence should be addressed, (JY, jyano@lbl.gov; MEW, mewall@lanl.gov)

SUPPORTING INFORMATION

Methodology

Supplementary Figures 1-19

Supplementary Tables 1-8

Supplementary References

1. Methodology

1.1 Mean structure factors (continued)

In order to deconvolute the MD density, individual water maps for selected channel waters were also computed. To do this, the expanded crystal structure in the P1 space group was used as a reference for the water positions in each copy. First, to make sure that the placement of each water was aligned for each of the four copies, the water of interest was selected across all copies and an Fcalc was computed just for the starting .pdb file. This map was then visualized to ensure that it only contained one peak, which confirmed that when the copies were folded back in on themselves, they overlapped. Next, in order to solely focus on dynamics that occurred in the 55 ns production run, a snapshot of the system immediately after equilibration (t = 45 ns) was taken. It is expected that during this 45 ns equilibration period, explicit waters will have rearranged themselves while still maintaining the overall water structure around the active site.

The updated water identities post-equilibration were then mapped to the reference positions. For example: If a new water "W_X" exchanges with W_{19} and fills the canonical W_{19} position during the 45 ns equilibration, then the residue id associated with "W_X" will be registered as W_{19} for the individual calculation. If no water at 45 ns is within 1 Angstrom of the canonical W_{19} position for a given copy, then no waters were selected for that copy when computing the average W_{19} map. Some of the individual maps which will be discussed are an average over all

four copies. Some are an average over only two copies, etc. These computed electron density maps were then used to determine the average positions of water molecules and identify regions of potentially high water mobility. Additionally, a short ion analysis was conducted by also computing a map over all Na^+ ions.

It is important to note that these maps were computed by averaging over all four dimers: the structure factors were computed by feeding in the complete trajectory which occurred in the MD simulations that were performed using a P1 unit cell, and the structure factor calculations were performed by specifying the $P2_12_12_1$ space group and folding all atoms back into the asymmetric unit. The result is a mean density for the $P2_12_12_1$ asymmetric unit. Strong peaks *(above a threshold of 2.08 e/Å^3 for water molecules and oxygen atoms, and above 0.26 e/Å^3 for hydrogen atoms - the factor of eight difference comes from the fact that hydrogen has 8x fewer electrons than oxygen)* were extracted from the simulated electron density maps using *CCP4* peakmax,[1] and their x,y,z coordinates were output into .pdb files.

For comparison between MD and experiment, *CCP4* MOLREP[1] was used to align the crystal structure to the MD density computed over the entire system. 133 waters were randomly selected outside of the channels for comparison.

1.2 Comparing MD map to Experimental map

In order to compare both the MD and experimental maps at a level which still preserved interesting water details, the following protocol was used. First, an absolute scale (electrons per Å^3) map was generated for the experimental 2Fo-Fc electron density. The absolute level of that display in electrons per Å^3 was noted at 1σ (0.173 e/Å^3). However, the full MD map contoured at the same level will not capture the shape and extent of the features we are interested in (aka, most individually resolved water densities). This is likely because the B-factors are quite low in the simulation, and the theoretical maxima of the electron distribution partially depends on the B-factor of the atoms. So, for visualization purposes, the complete MD map was adjusted by-eye in multiples of 0.173 until their contour roughly matched that of the experimental map in several regions which are believed to be rigidly fixed (**ARG 320 C**, **HIS 337 A**, **PHE 317 C**) (see **Supplementary Figure 1**). All MD maps are shown at a level of 2.08 e/Å^3 unless explicitly stated otherwise for visualization purposes. Any experimental maps are shown at 0.173 e/Å^3 (equivalent to 1σ).

1.3 MADI: Map-based donor-acceptor identification

In order to infer about PS II's hydrogen bonding networks, a new method called MADI (map-based donor-acceptor identification) was deployed. The method was developed to evaluate average hydrogen bond strength and direction between neighboring waters over the course of the trajectory: First, strong peaks (above a threshold of 2σ) were extracted from the following three MD simulation electron density maps using *CCP4* peakmax,[1] and their x,y,z coordinates were output into .pdb files.

1) A map computed using all water oxygen atoms
2) A map computed using the first hydrogen atom in all waters *(HW1 only)*
3) A map computed using the second hydrogen atom in all waters *(HW2 only)*

Next, the list of peaks from the water-oxygen map was looped through. If an oxygen peak was found within 1.5 Å of a crystallographic water, then that peak would be grouped with that water and labeled as that water's "average water-oxygen position" over the course of the simulation. The two hydrogen-peak lists were looped through next. For each list, if a hydrogen peak was found to be within 1.4 Å of the average water-oxygen position for a crystallographic water, then the hydrogen peak would also be "grouped" into that water position. The end result ideally involved one average oxygen and two average hydrogen peaks per crystallographic water (when looking at the 100 waters of focus, we identified 1.7 hydrogens per water on average) - leading to an average water orientation for every crystallographic water over the course of the simulation. The above procedure yielded putative MD water orientations for many, but not all, of the crystallographic waters. Optimal O-H bonds were determined and classified by strength (see **Supplementary Figures 17** and **18).**

To avoid pitfalls in interpretation, it was important to apply several layers of filtering. Simply looping through, checking average oxygen-hydrogen distances between nearest neighbors, and considering it as satisfactory when an O-H distance was under a given threshold – could bury important orientational information regarding the direction of the bond and the identity of acceptor vs donor. See **Supplementary Figure 19** for a flowchart which details the filtering logic employed in the hydrogen-bonding analysis.

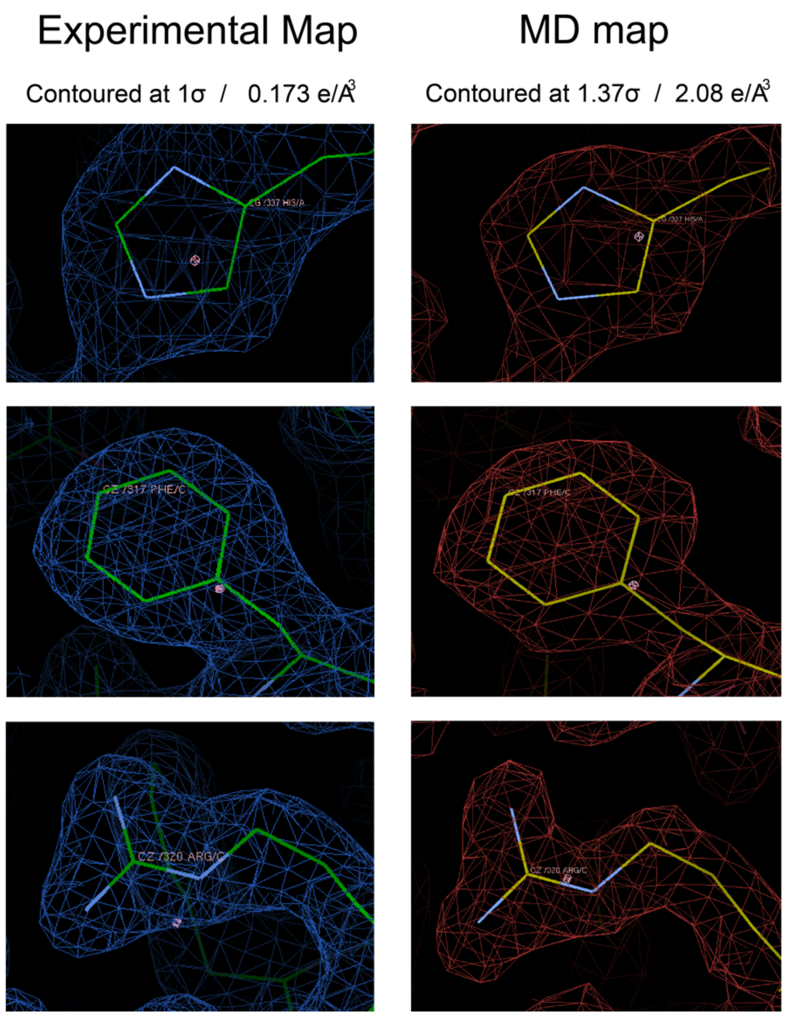

Supplementary Figure 1. *Converging to comparable maps between the experimental 2Fo-Fc crystallographic densities (left, blue, contoured at 0.173 e/$Å^3$ - which is equivalent to 1σ), and the MD densities (right, contoured at 2.08 e/$Å^3$). Images shown in Coot.*[2]

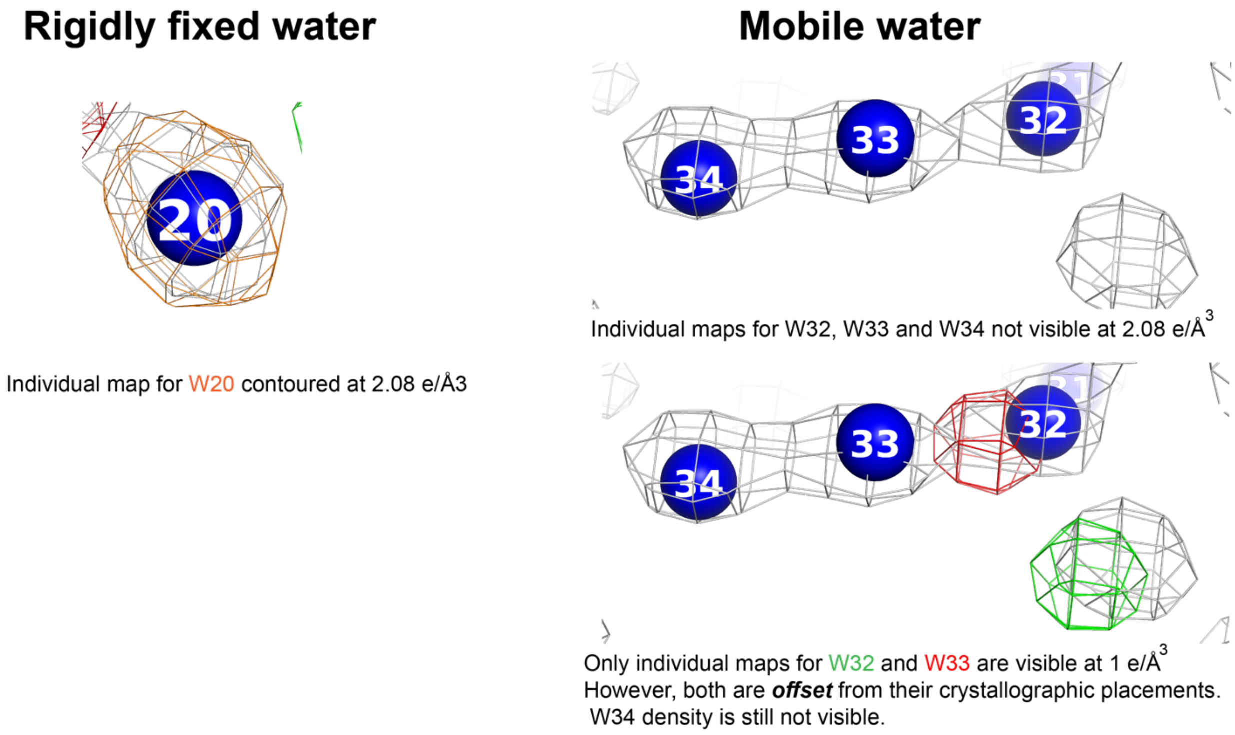

Supplementary Figure 2. *Individually calculated water maps (colored) overlaid with the total average water map across all copies (grey) to demonstrate how mobility is classified. (Left) Example of how the individual water map looks for a rigidly fixed water. W20's individual map, which shows up as a visible and strong sphere of density, is comparable to the total water map when both are at the same 2.08 e/Å^3 level. (Right) On the other hand, the individual maps for more mobile waters like W32-W33-W34 are not visible at 2.08 e/Å^3, since they are not in any location long enough over the course of the simulation to build up substantial density. At 1 e/Å^3, individual maps for W32 and W33 become visible (and are offset from their crystallographic placements, with the density for W33 sitting closer to W32 and the density for W32 sitting in the W39 position). The density for W34, on the other hand, remains still too weak to be seen at this level due to extreme mobility. It only becomes visible at levels < 0.7 e/Å^3.*

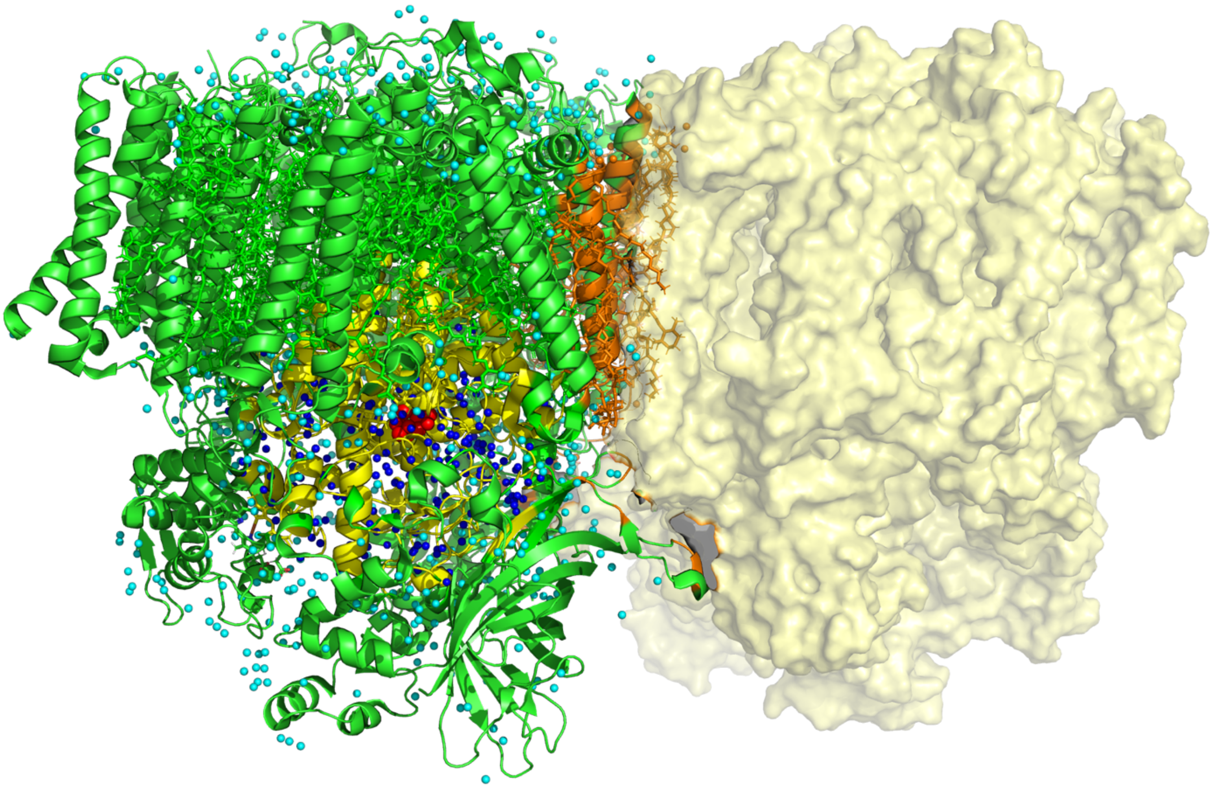

Supplementary Figure 3. *The full PS II dimer (PDB ID 7**RF2**) which was used as the basis for our MD model. The ROI referred to throughout the text (centered on the O1 atom in the OEC with a 25 Å radius) is shown (yellow) along with its enclosed waters (dark blue). Note that the orientation of the dimer is flipped relative to how it is displayed in **Figure 1**. All outside waters are shown in cyan. The 'unlocked' monomer,*[3] *which is the focus of this study, is shown in green. The 'locked' monomer*[3] *is shown as a sand-colored surface. The orange region corresponds to contacts within 3.5 Å of the monomer-monomer interface extended to the entire residue/ligand.*

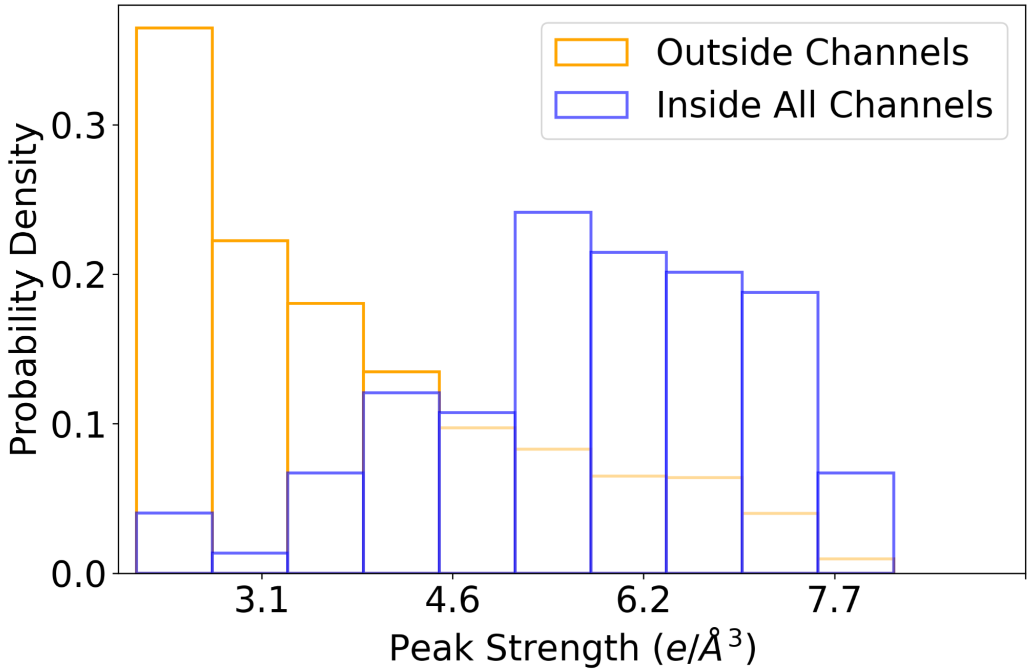

Supplementary Figure 4. *Histogram comparing the distribution of peak height of simulated waters inside the three channels of interest (O1, Cl and O4) vs. outside the channels. On average, waters inside the channels demonstrated overall stronger peaks. Such a finding suggests that positioning of waters within the channels is more anchored over the course of the simulation. Each bin corresponds to raw count divided by the total number of counts as well as the bin width. As a result, the area under each histogram is normalized to one – thus providing a probability density.*

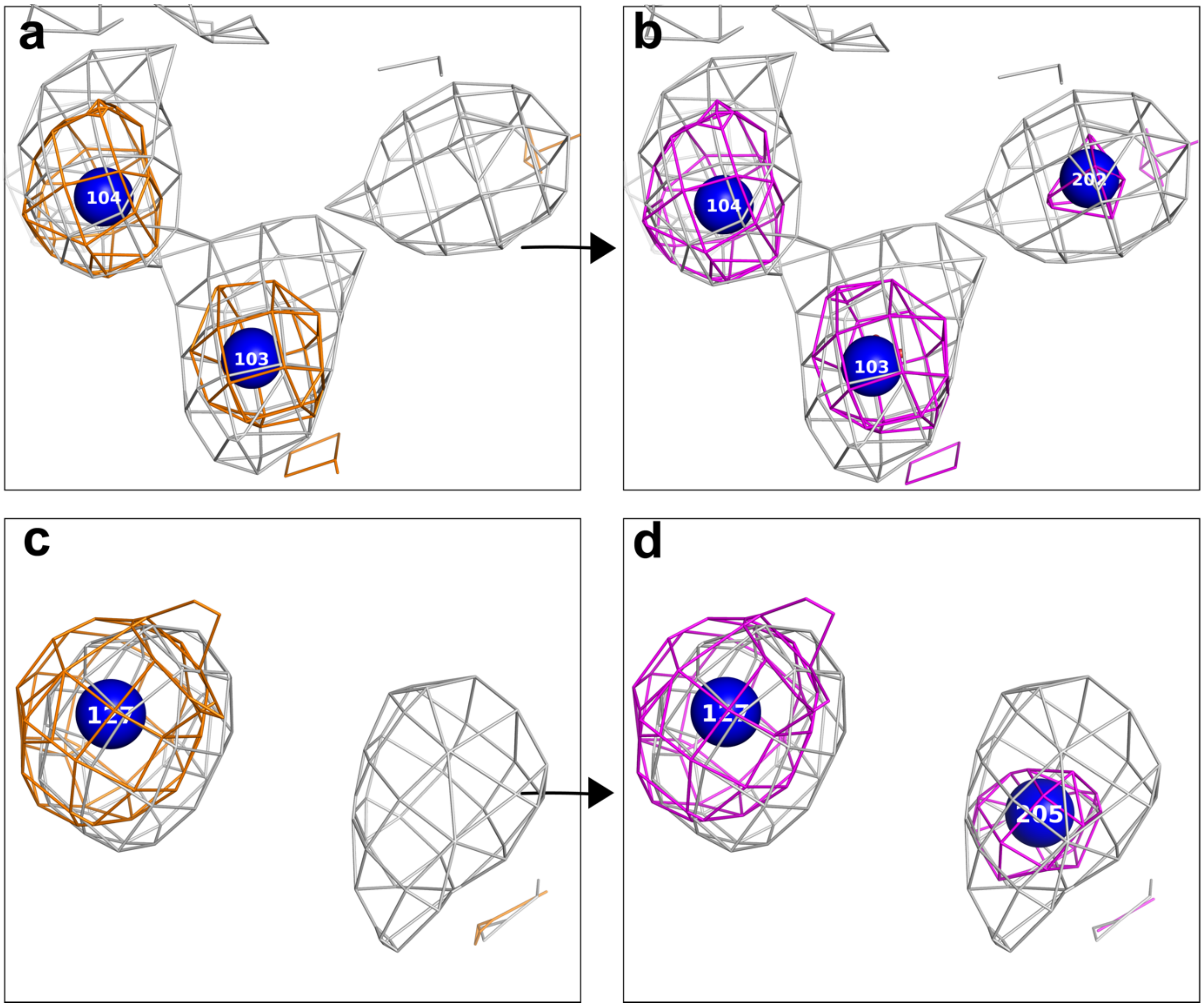

Supplementary Figure 5. *Demonstration of how crystallographic refinement can be improved through the combination of MD. **(a)** MD solvent map (grey and contoured at 2.08 e/Å^3) overlaid with the experimental 2Fo-Fc map (orange and contoured at 1 σ). **(b)** After an additional round of MD-aided refinement, a new water, W202, was registered in this region (updated 2Fo-Fc map is colored magenta and contoured at 1σ). **(c)** An additional MD solvent density, not found in the experimental map, appears near W127. **(d)** After an additional round of MD-aided refinement, yet another new water, W205, was registered in this region.*

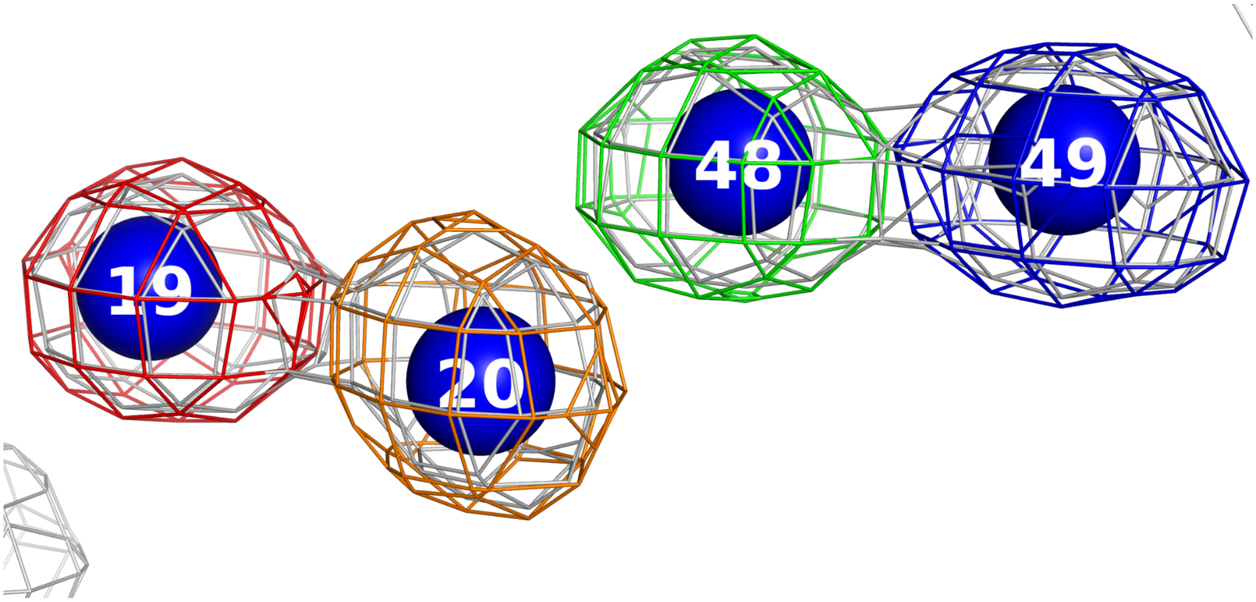

Supplementary Figure 6. *Individually calculated water maps (colored) overlaid with the total water map (grey) in the O4 channel. All contoured at 2.08 e/Å^3.*

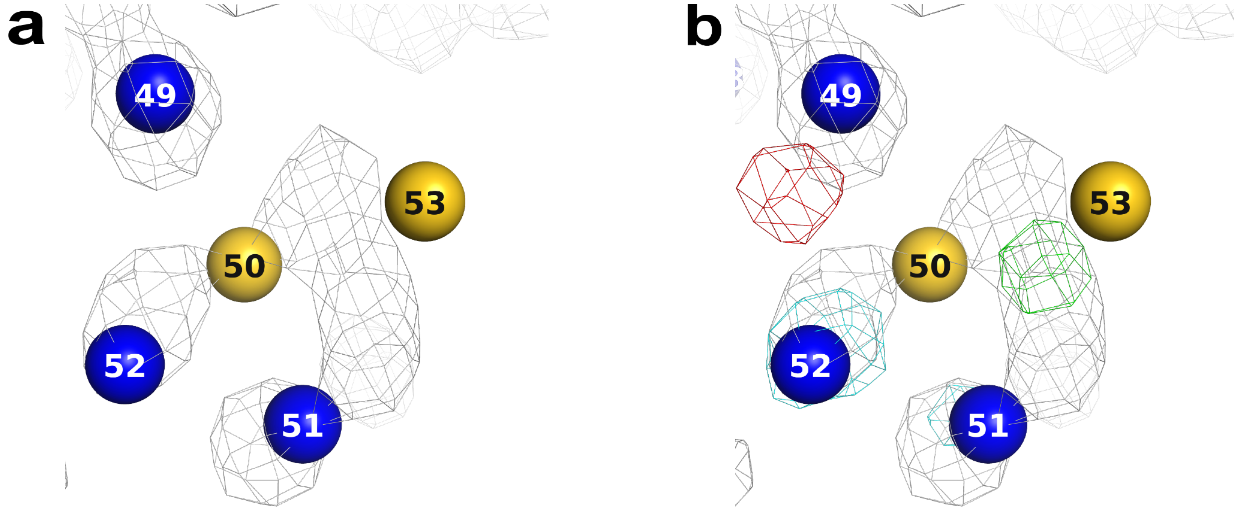

Supplementary Figure 7. *Individually calculated water maps (colored) in the region of W50-W51-W52-W53 overlaid with the total water map (grey).* ***(a)*** *All maps contoured at 2.08 e/Å^3, yet only the full water map is visible at this level.* ***(b)*** *Individual maps for W50 (red), W51 (green) and W52 (cyan) contoured at 1 e/Å^3 for better visualization, while the total water map remains contoured at 2.08 e/Å^3. Dynamic tendencies are observed in this region. Note that no individual map was calculated for W53 as there was no equivalent water near its position after the 45 ns equilibration (see Methods).*

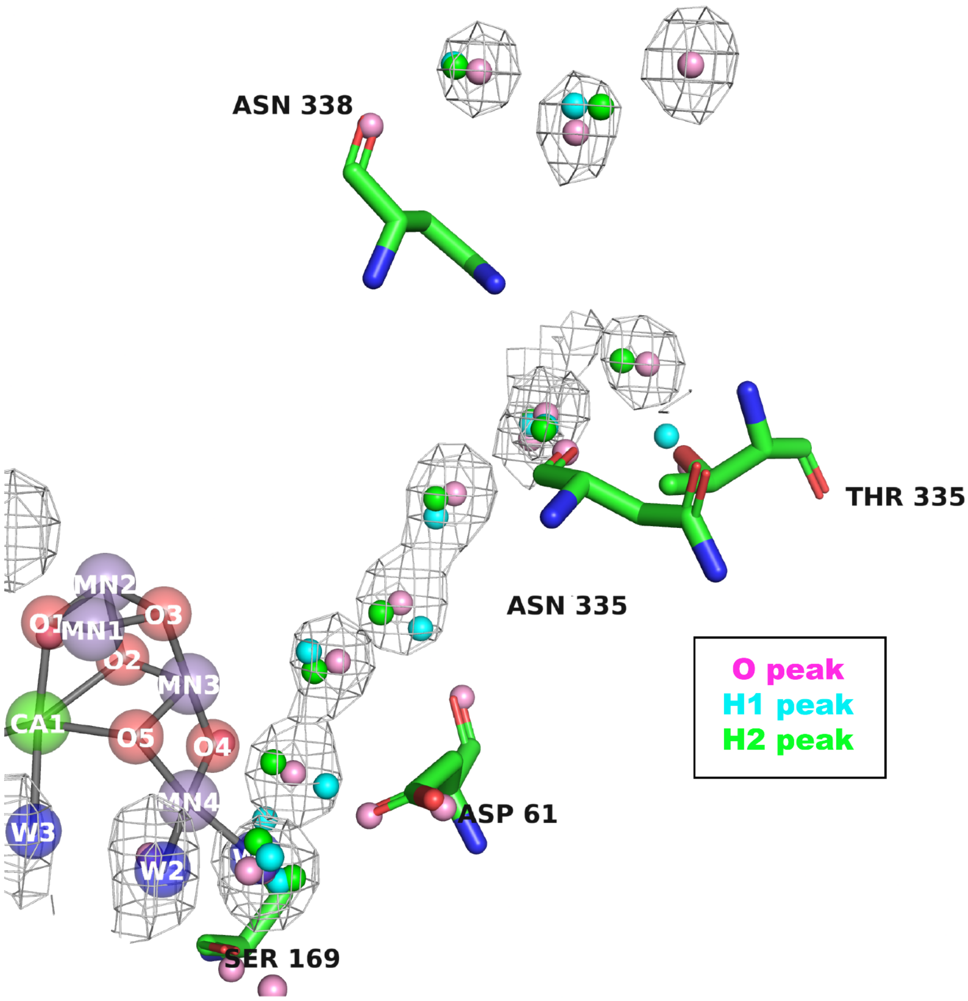

Supplementary Figure 8. *Oxygen and hydrogen peaks from the MADI analysis - demonstrating the average orientation of waters which are hypothesized to participate in bonding in the O4 channel.*

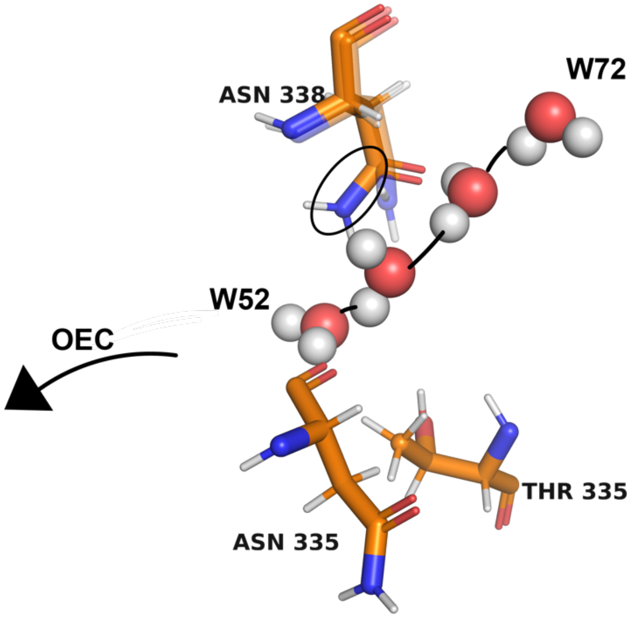

Supplementary Figure 9. *Snapshot at 45ns (third dimer, monomer A, for details see **Methods**), at which point an H-bond chain transiently bridges the gap between the W50-W51-W52-W53 region and the W71-W72-W73 triangle. The ASN sidechain appears also to bend into a conformation which maximizes space for the transiting water. The resting state structure for ASN 338 is overlaid at a higher transparency to demonstrate ASN movement.*

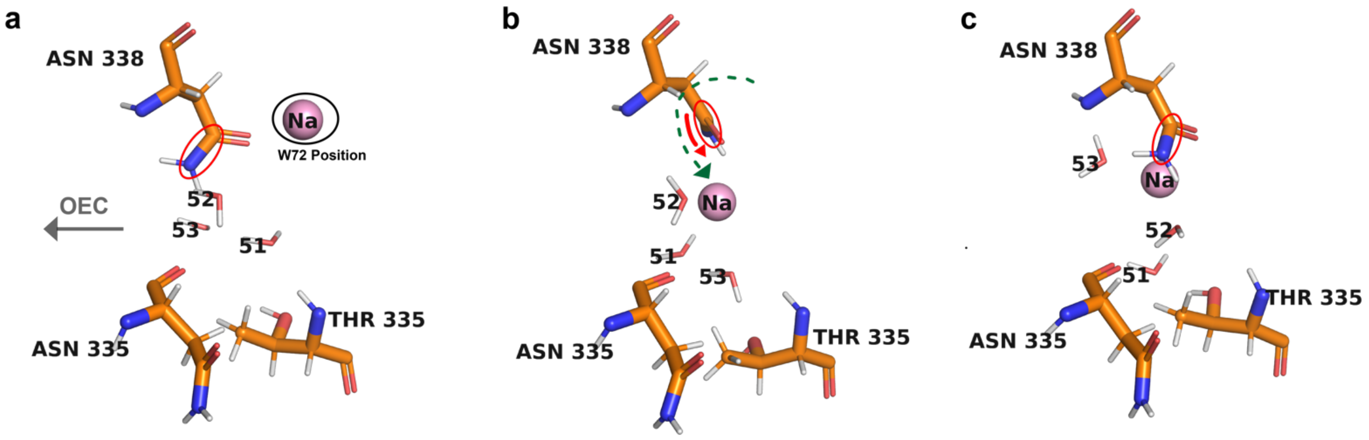

Supplementary Figure 10. *Schematic of the ASN drawbridge mechanism observed at 0 ns* ***(a)****, 0.28 ns* ***(b)*** *and 2.74 ns* ***(c)*** *during the equilibration period (second dimer, monomer A, for details see* ***Methods****). In this example, the twisting of the ASN sidechain makes room for a* Na^+ *ion hopping from the W72 position into a region near W51-W52-W53.*

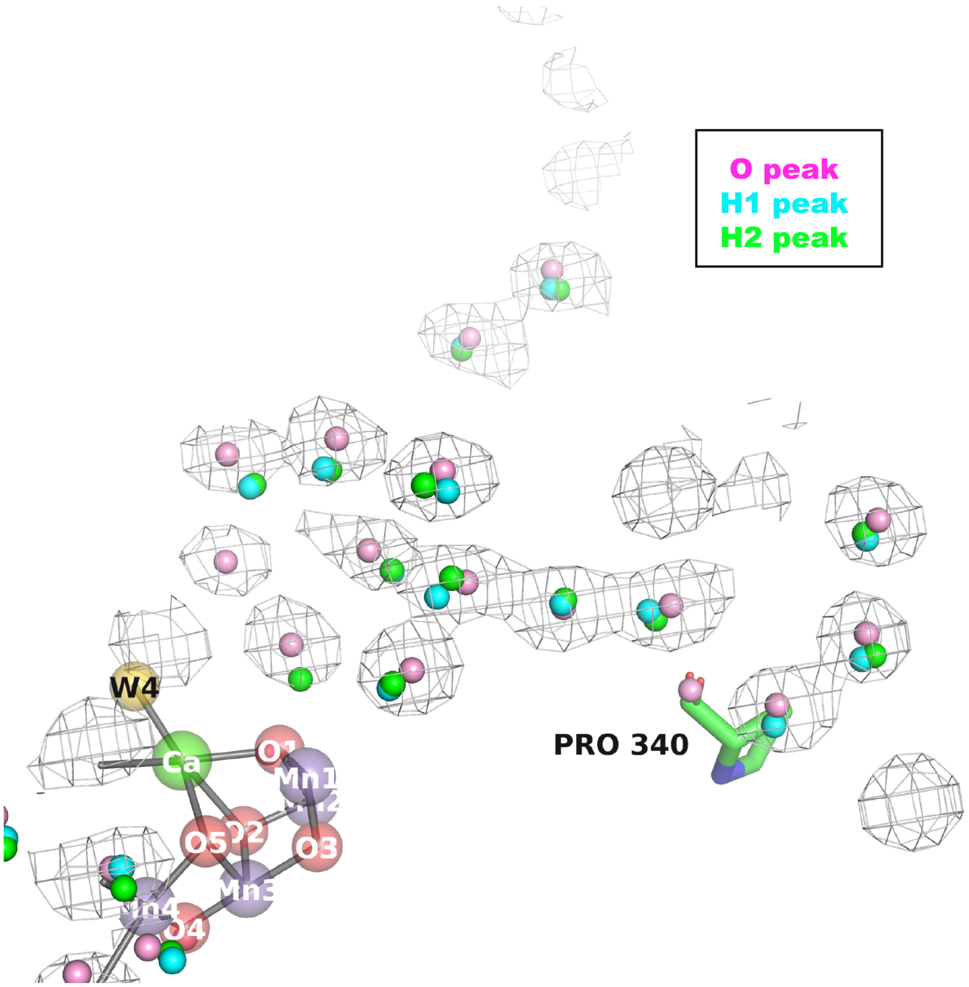

Supplementary Figure 11. *Oxygen and hydrogen peaks from the MADI analysis - demonstrating the average orientation of waters which are hypothesized to participate in bonding in the O1 channel.*

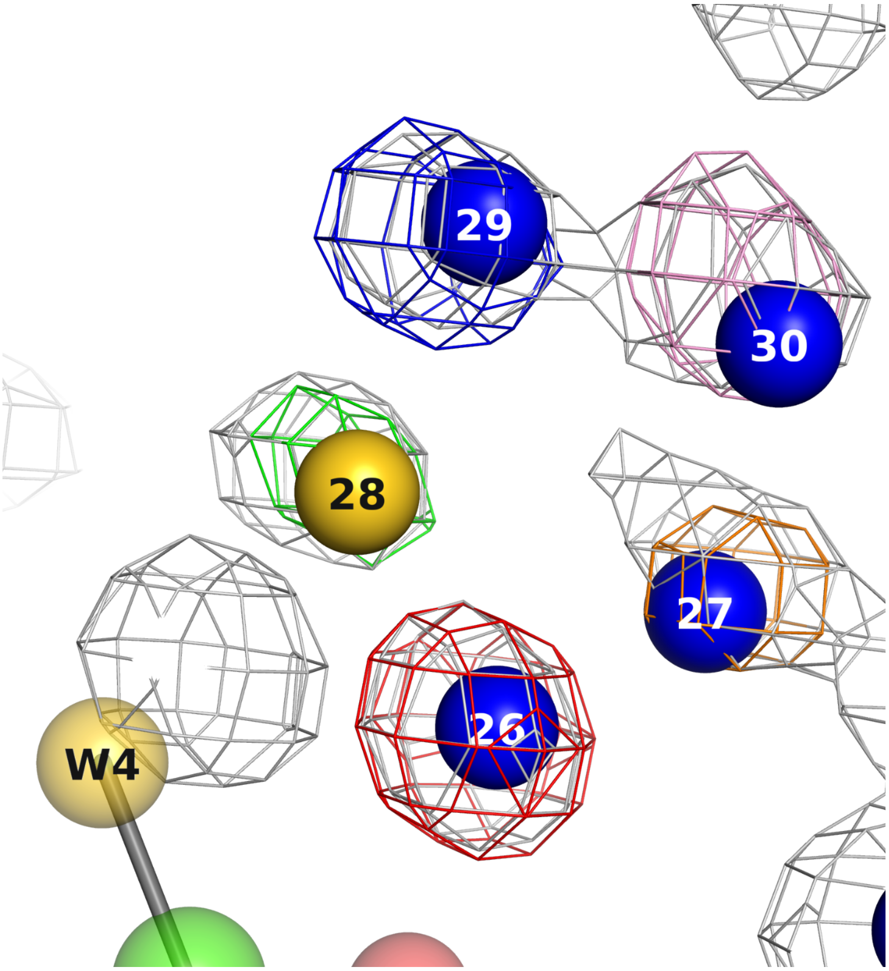

Supplementary Figure 12. *Calculated water maps for individual waters (colored) in the penta-cluster (or water wheel), overlaid with the total water map (grey). Maps contoured at 2.08 e/Å^3. Note that all individual water maps are visible at this level. The grey density next to W4 corresponds to an offset W4 position during the simulation.*

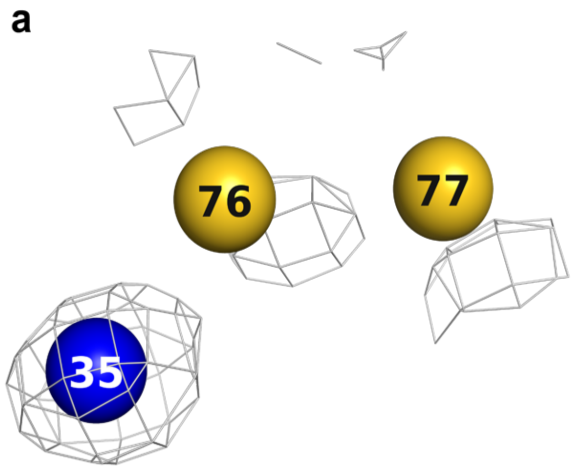

Supplementary Figure 13. *Calculated individual water maps for W35-W76-W77 overlaid with the total water map (grey).* ***(a)*** *All maps contoured at 2.08 e/Å^3, yet only the total water map (grey) is visible at this level.* ***(b)*** *Individual water maps for W35 and W77 contoured at 1 e/Å^3 for better visualization, yet only the map for W35 (red) is visible. Note that no individual map was calculated for W76 as there was no equivalent water near its position after the 45 ns equilibration (see Methods). The density for W77 only becomes visible at levels < 0.55 e/Å^3.*

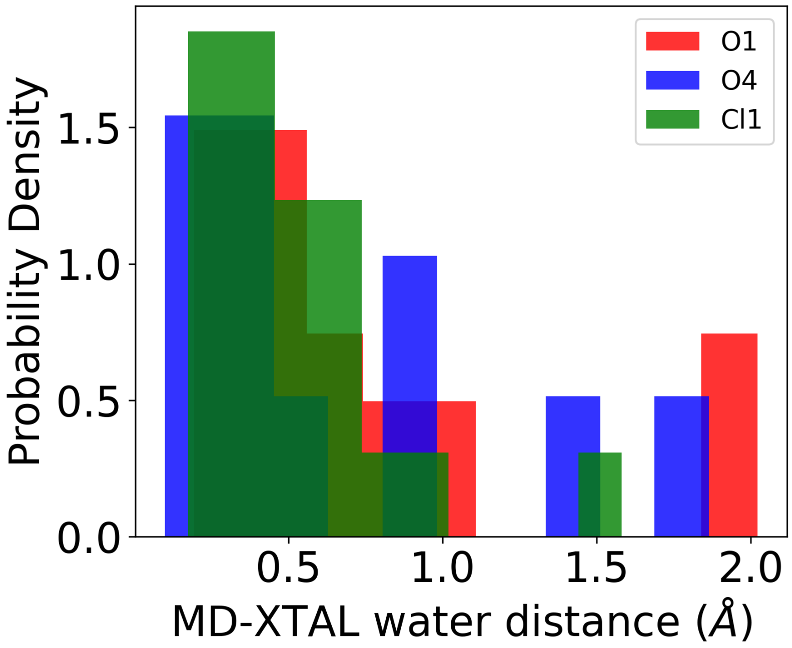

Supplementary Figure 14. *Distribution of the distances between crystallographic water positions and the nearest strong MD peaks (>2.08 e/Å^3) within three channels of interest. Each bin corresponds to raw count divided by the total number of counts in each channel as well as the bin width. As a result, the area under each histogram is normalized to one – thus providing a probability density.*

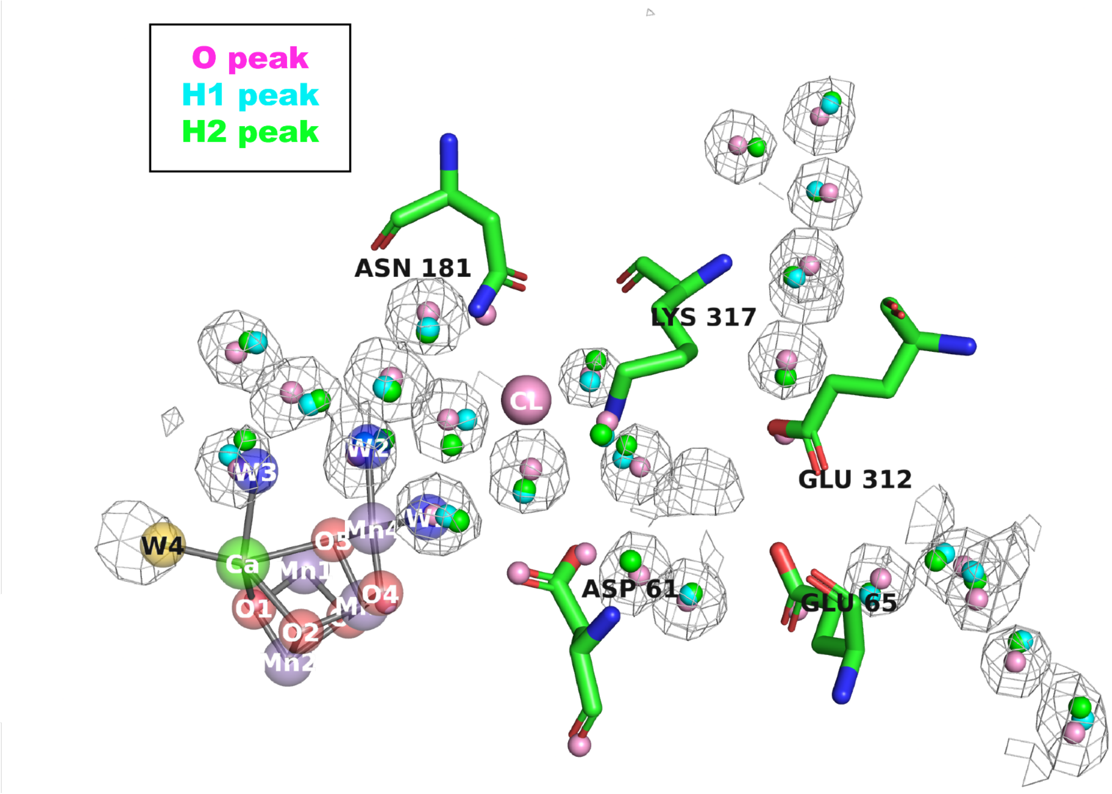

Supplementary Figure 15. *Oxygen and hydrogen peaks from the MADI analysis - demonstrating the average orientation of waters which are hypothesized to participate in bonding in the Cl1 channel.*

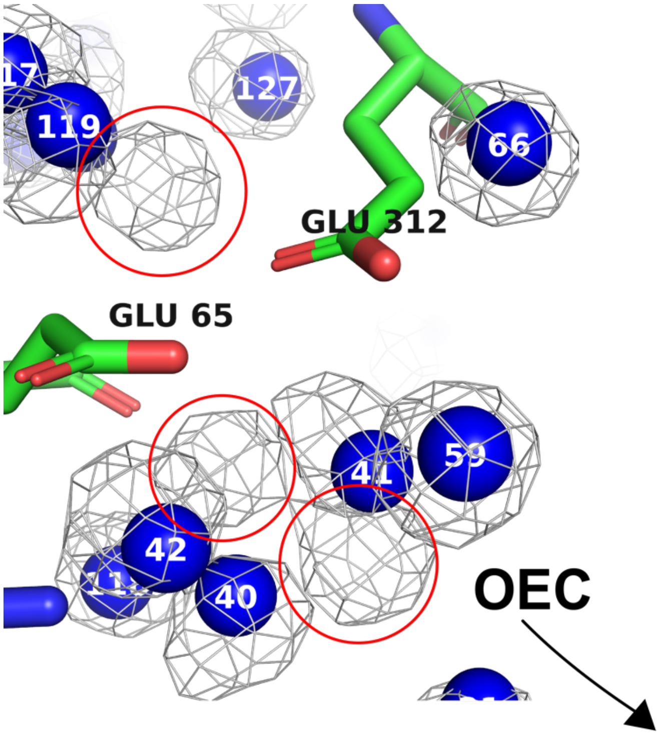

Supplementary Figure 16. *Three extra densities observed on both sides of the GLU 65- GLU 312 'proton gate' in the Cl1 channel. Solvent map contoured at 2.08 e/Å^3 and overlaid with the crystal structure. In the crystal structure, the GLU 65 and GLU 312 distance is 2.7 Å. In the final snapshot from the MD trajectory, the GLU 65 and GLU 312 distance is at least 3 Å across all four copies. We hypothesize that these additional water positions can only be occupied when the bottleneck is wider than in the XRD structure.*

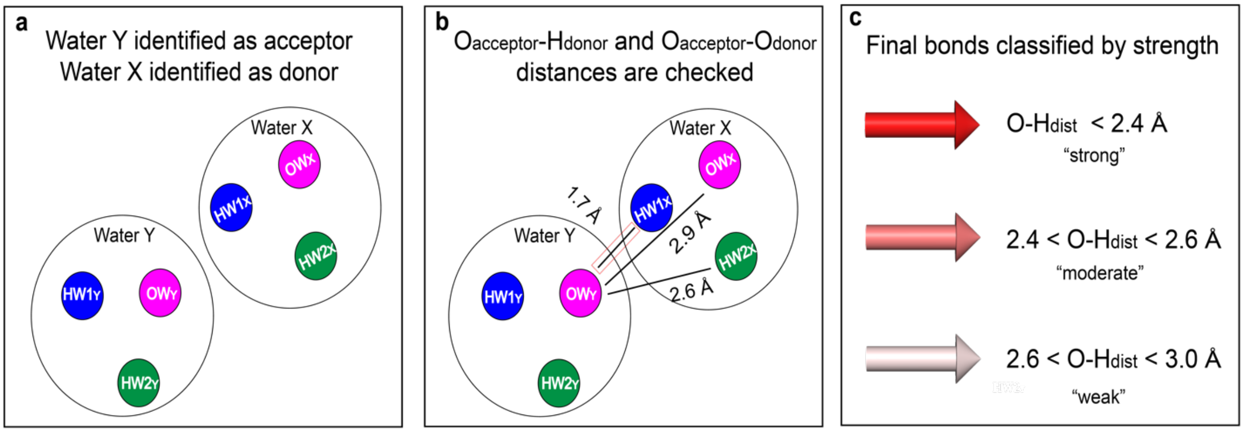

Supplementary Figure 17: *Determining optimal O-H bonds. (A) After oxygen and hydrogen peaks are grouped, forming an average water orientation from the simulation, all combinations of O-H peak distances between nearest neighbors were checked in order to converge on the optimal donor-acceptor direction for a given pair. From panel A, water Y is clearly in a more optimal configuration for accepting a proton from water X than the other way around. (B) Once acceptor and donor roles were established, a few strict thresholds were set to start filtering the results. O-H distances had to be below 3.0 Å, and* O_{donor}*-* $H_{acceptor}$ *distance <* O_{donor}*-* $O_{acceptor}$ *distance. The latter filter was put in place to ensure that the orientation between donor-acceptor pairs was optimized for hydrogen bonding. While both* OW_y *-* $HW1_x$ *and* OW_y *-* $HW2_x$ *from panel B are feasible bonds, the distance of the former is smaller, so it is selected as the optimal O-H bond distance in the pairing. After all optimal bonds are identified between pairs, if a water is registered as participating as a "donor" in three surviving bonds, the weakest of the three is knocked out. (C) The strength of the final bonds were classified based on the distribution of O-H distances retrieved from the MADI analysis (see* **SI 18***). The results are represented by arrows following a color gradient, pointing from donor to acceptor (see* ***Results****).*

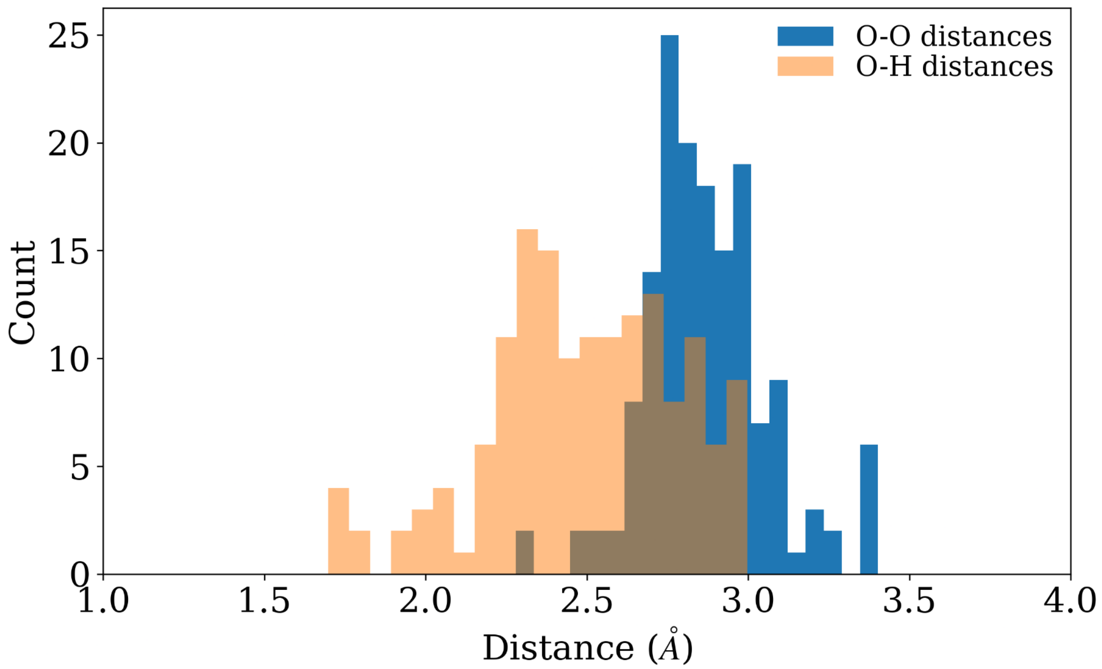

Supplementary Figure 18. *Distribution of O-O and O-H distances retrieved from the MADI analysis. For the O-H distribution the 33rd, 66th and 99th percentile are at 2.4 Å, 2.6 Å, 3.0 Å, respectively.*

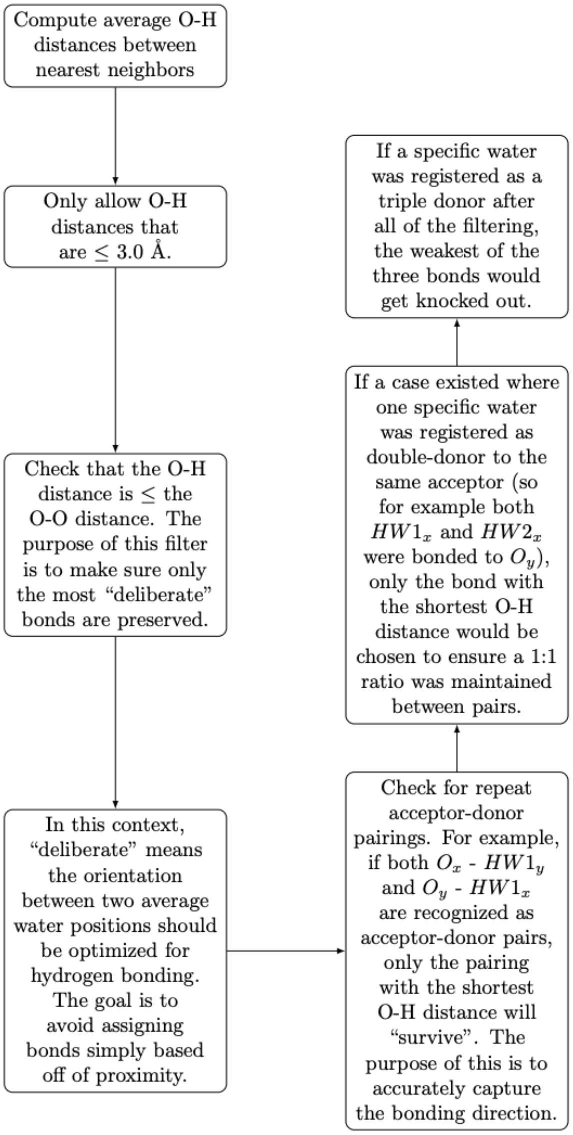

Supplementary Figure 19. *Flowchart detailing the filtering logic employed for the hydrogen-bonding analysis.*

Supplementary Table 1. *Breakdown of lipids/cofactors/ions used in simulation*

Lipid/Cofactor/Ion	Copies in Simulation
OXYGEN EVOLVING COMPLEX	*8*
PHEOPHYTIN A	*16*
1,2-DI-O-ACYL-3-O-[6-DEOXY-6-SULFO-ALPHA-D-GLUCOPYRANOSYL]-SN-GLYCEROL (SQDG)	*32*
PROTOPORPHYRIN IX CONTAINING FE (Heme)	*16*
1,2-DIPALMITOYL-PHOSPHATIDYL-GLYCEROLE (PG)	*40*
1,2-DISTEAROYL-MONOGALACTOSYL-DIGLYCERIDE (MGDG)	*52*
2,3-DIMETHYL-5-(3,7,11,15,19,23,27,31,35-NONAMETHYL-2,6,10,14,18,22,26,30,34-HEXATRIACONTANONAENYL-2,5-CYCLOHEXADIENE-1,4-DIONE-2,3-DIMETHYL-5-SOLANESYL-1,4-BENZOQUINONE (PQ)	*16*
BETA-CAROTENE (Car)	*86*
CHLOROPHYLL A	*280*
DIGALACTOSYL DIACYL GLYCEROL (DGDG)	*40*
STEARIC ACID	*124*

FE (II) ION	*8*
CHLORIDE ION	*16*
W1, W2, W3 W4	*8*

.

Supplementary Table 2. *Breakdown of atoms in simulation*

Element	# Atoms
Protein	330432
Crystal Waters	25260
Solvent molecules added by GROMACS	424731
Ligands/lipids/cofactors/ions	81471

Supplementary Table 3. *Documentation of how various lipids/cofactors/ions were prepared for this study.*

Ligand	Mol2 Source	Frcmod Source	Hydrogen Source
PHO	Sakashita	Sakashita	Tleap
BCR	Sakashita	Sakashita	Tleap
PL9	Sakashita	Sakashita	Tleap
HEM	Sakashita	Sakashita	Tleap

CLA	Sakashita	Sakashita	Tleap
SQD	This study	This study	Tleap
DGD	Sakashita	This study	Tleap
STE	This study	This study	Tleap
LMG	Sakashita	This study	Tleap
LHG	Sakashita	Sakashita	Tleap
FE2	This study	This study	N/A
CL	This study	This study	N/A

Supplementary Table 4. *Net charges for the lipids/cofactors/ions used in this simulation.*

Residue	Net Charge
PHO	0
BCR	0
PL9	0
HEM	-2
CLA	0
SQD	0
DGD	0
STE	-1
LMG	0

LHG	-1
FE2	+2
CL	-1

Supplementary Table 5. *All significant water wires (must contain ≥ 3 waters with a consistent acceptor-donor orientation) identified by the MADI analysis.*

Channel	Constituents	Orientation
**O4**	W49-W48-W20-O4<br>W49-W48-W20-W19-O4<br>W49-W48-W20-W19-**ASP 61**	Towards active site
**O1**	W29-W30-W27-W32-W31<br>W29-W30-W27-W32-W39	Away from active site
**O1**	W34-W33-W32-W31<br>W34-W33-W32-W39	Towards active site
**O1**	W38-W37-W36	Towards active site
**Cl1**	W3-W23-W24-W2-W22-**ASN 181**<br>W3-W23-W24-W2-W22-W21-**ASP 61**<br>W3-W23-W24-W2-W22-W21-W1-**ASP 61**	Away from active site
**Cl1**	W121-W117-W119	Towards active site
**Cl1**	W126-W122-W117-W119	Towards active site

Supplementary Table 6. *Defining O1 channel by amino acids. ROI is 3.5 Å for all waters.*

Water	Amino	Amino	Amino	Amino	Amino	Amino	Amino	Amino

	acid atom name #1	acid resn #1	acid chain #1	acid resid #1	acid atom name #2	acid #2	acid chain #2	acid resid #2
103	O	ALA	C	399	N	GLY	C	409
104	O	THR	C	397	N	GLY	C	409
106	CG	GLU	C	83	N	ASN	C	418
107	C	GLU	C	83	O	ASN	C	418
109	O	GLN	V	34	N	ASN	C	418
26	CD	GLU	A	189	C	ALA	A	344
27	O	ASP	A	342	CA	GLU	A	189
29	O	GLU	A	189	OD1	ASN	A	296
31	O	ALA	A	188	OE1	GLU	A	329
32	OE1	GLU	A	329	O	ASP	A	342
33	N	ASP	A	342	OE2	GLU	A	329
34	O	PRO	A	340	NZ	LYS	V	134
36	O	PRO	A	340	NZ	LYS	V	134
37	N	LYS	U	104	NZ	LYS	V	134
38	CB	LYS	U	104	CB	LYS	V	134
101	NH1	ARG	B	384	N	TYR	U	103
102	NH1	ARG	B	384	OE2	GLU	B	387

Supplementary Table 7. *Defining Cl1 channel by amino acids. ROI is 3.5 Å for all waters except for W40, W121, W24 and W61, which have a radius of 5 Å.*

Water	Amino acid atom name #1	Amino acid resn #1	Amino acid chain #1	Amino acid resid #1	Amino acid atom name #2	Amino Acid resn #2	Amino acid chain #2	Amino acid resid #2
21	OD1	ASP	A	61	NZ	LYS	D	317
40	OE2	GLU	D	312	OD2	ASP	A	61
42	OE1	GLU	A	65	N	ARG	A	334
119	CD	GLU	A	65	C	ASP	O	158
117	O	PRO	D	309	CG	GLU	A	65
121	NH2	ARG	O	152	O	GLU	D	310
125	O	PRO	D	309	OD1	ASP	O	224
126	OE1	GLU	D	310	NH2	ARG	O	152
140	O	ARG	A	64	CG	PRO	O	117
143	CG	PRO	O	117	OH	TYR	A	107
25	OH	TYR	A	161	CD	GLU	A	189
24	CA	ASP	A	170	N	PHE	A	186
61	CA	PHE	A	182	O	SER	A	169
22	ND2	ASN	A	181	OE2	GLU	A	333

60	O	MET	A	331	O	LYS	D	317
66	CD	GLU	D	312	O	PRO	O	159
67	CB	LEU	D	319	N	LYS	O	160
68	OE1	GLU	D	323	CA	LYS	O	160
69	OE1	GLU	D	323	NZ	LYS	O	160
130	OH	TYR	D	296	NH2	ARG	D	326
132	O	GLU	B	364	O	ILE	O	172
133	OD2	ASP	D	297	O	ALA	O	173
135	NH1	ARG	B	326	CG2	ILE	B	442
136	NZ	LYS	B	321	O	GLU	B	364
137	O	LYS	B	321	NH1	ARG	B	326

Supplementary Table 8. *Defining O4 channel by amino acids. ROI is 3.5 Å for all waters.*

Water	Amino acid atom name #1	Amino acid resn #1	Amino acid chain #1	Amino acid resid #1	Amino acid atom name #2	Amino acid resn #2	Amino acid chain #2	Amino acid resid #2
19	OD1	ASP	A	61	NH2	ARG	C	357
20	OE2	GLU	C	354	CB	ASP	A	61

48	O	ASP	A	61	ND2	ASN	A	87
49	CG2	ILE	A	63	C	GLY	C	353
50	OG1	THR	C	335	CA	GLY	C	353
51	OG1	THR	C	335	O	LEU	C	337
52	O	ASN	A	335	ND2	ASN	A	338
53	O	GLY	C	338	SD	MET	C	342
71	O	ASN	A	338	OD1	ASN	U	99
72	CD	PRO	C	334	ND2	ASN	D	350
73	ND2	ASN	O	155	OD1	ASP	U	96
74	O	GLN	C	332	OG1	THR	O	153
75	N	ASN	O	155	OD2	ASP	U	96
113	O	ALA	U	90	OD2	ASP	U	100
114	OD1	ASN	O	155	O	ASN	U	100
112	CB	ASN	U	31	ND2	ASN	U	100
115	OH	TYR	U	21	N	ASN	U	31

Supplementary Table 9. *Number of MD peaks and crystallographic waters found in each of the three channels, using the amino acid boundaries tabulated in supplementary tables 5-7.*

**Channel**	**Number of MD peaks**	**Number of Crystallographic Waters**

O1	30	22
O4	20	19
Cl1	46	35

Supplementary References

(1) Winn, M. D.; Ballard, C. C.; Cowtan, K. D.; Dodson, E. J.; Emsley, P.; Evans, P. R.; Keegan, R. M.; Krissinel, E. B.; Leslie, A. G. W.; McCoy, A.; McNicholas, S. J.; Murshudov, G. N.; Pannu, N. S.; Potterton, E. A.; Powell, H. R.; Read, R. J.; Vagin, A.; Wilson, K. S. Overview of the *CCP4* Suite and Current Developments. *Acta Crystallogr. D Biol. Crystallogr.* **2011**, *67* (4), 235–242. https://doi.org/10.1107/S0907444910045749.

(2) Emsley, P.; Lohkamp, B.; Scott, W. G.; Cowtan, K. Features and Development of *Coot*. *Acta Crystallogr. D Biol. Crystallogr.* **2010**, *66* (4), 486–501. https://doi.org/10.1107/S0907444910007493.

(3) Zhang, M.; Bommer, M.; Chatterjee, R.; Hussein, R.; Yano, J.; Dau, H.; Kern, J.; Dobbek, H.; Zouni, A. Structural Insights into the Light-Driven Auto-Assembly Process of the Water-Oxidizing Mn4CaO5-Cluster in Photosystem II. *eLife* **2017**, *6*, e26933. https://doi.org/10.7554/eLife.26933.
